# Neuraminidase as a novel therapeutic management strategy for Alzheimer’s disease: evidenced through molecular docking, molecular dynamic simulation and gene expression analysis

**DOI:** 10.3389/fchem.2025.1574702

**Published:** 2025-05-30

**Authors:** Sami I. Alzarea, Omar Awad Alsaidan, Hassan H. Alhassan, Abdulaziz Ibrahim Alzarea, Tariq G. Alsahli, Metab Alharbi, Muhammad Afzal, Mohammad Jaffar Sadiq Mantargi

**Affiliations:** ^1^ Department of Pharmacology, College of Pharmacy, Jouf University, Sakaka, Al-Jouf, Saudi Arabia; ^2^ King Salman Centre for Disability Research, Riyadh, Saudi Arabia; ^3^ Department of Pharmaceutics, College of Pharmacy, Jouf University, Sakaka, Al-Jouf, Saudi Arabia; ^4^ Department of Clinical Laboratory Sciences, College of Applied Medical Sciences, Jouf University, Sakaka, Al-Jouf, Saudi Arabia; ^5^ Department of Clinical Pharmacy, College of Pharmacy, Jouf University, Sakaka, Al-Jouf, Saudi Arabia; ^6^ Department of Pharmacology and Toxicology, College of Pharmacy, King Saud University, Riyadh, Saudi Arabia; ^7^ Department of Pharmaceutical Sciences, Pharmacy Program, Batterjee Medical College, Jeddah, Saudi Arabia

**Keywords:** Alzheimer’s disease, oseltamivir, neuraminidase, molecular docking, molecular dynamic simulation, gene expression analysis, gene enrichment analysis, insilico study

## Abstract

**Introduction:**

Neuraminidase in humans is studied to see how well repurposed oseltamivir works for treating Alzheimer’s disease (AD) using methods like molecular docking, molecular dynamic (MD) simulation, and gene expression analysis. Gene enrichment analysis was also studied to understand the behaviour of neuraminidases in humans.

**Methods:**

Molecular docking was done using oseltamivir and the neuraminidase proteins with the *PyRx* tool, and the results were analysed using BIOVIA Discovery Studio. MD simulation (50 ns) of the oseltamivir and neuraminidase complex was performed using GROMACS tools. The gene expression analysis and gene enrichment study were done using GEO2R, which showed the results as log FC and significant values. Enricher tool-based gene enrichment analysis was done to determine the gene behaviour related to the AD.

**Results:**

The molecular docking showed a strong connection between oseltamivir and neuraminidase (−6.5 kcal/mol), acetylcholinesterase (−7.9 kcal/mol), CDKs (−6.5 kcal/mol), and GSKs (−6.6 kcal/mol), interacting with different amino acids in the protein sequences. MD simulations showed a strong interaction between the ligand and neuraminidase, with stable measurements indicating that both the protein and ligand remained consistent in size and energy, which is better explained through the results of MM_PBSA and MM_GBSA analysis of the complex, resulting in the ΔE_vdW, ΔE_elec, ΔG_polar, ΔG_nonpolar, ΔG_gas, (ΔE_vdW + ΔEEL), ΔG_solvation: (ΔG_polar + ΔG_nonpolar) and ΔG_bind: total energies suggesting the complex stayed stable in conditions similar to those resembling natural cell. The gene expression analysis expressed TUBB3 (formation of beta-tubulin), FABP3 (regulates alpha-synuclein uptake in dopaminergic neurons), and CALM1 (calcium signal transduction pathway) to be highly upregulated in the given conditions with kinase binding (p = 0.0006541) and protein phosphatase regulatory activity (p = 0.001357) were highly upregulated, implicating their importance in the AD.

**Discussion:**

The study ends on a hopeful note for using oseltamivir to treat neurological diseases, but it suggests that future research should include a solid cell line study, an *in vitro* study, and a clinical study.

## Introduction

Alois Alzheimer pioneered the study of memory loss. He observed the existence of amyloid plaques and an immense harm to neurons during brain examinations of his very first patient who was suffering from cognitive loss and personality change before the patient died. Alois Alzheimer defined this particular condition as a disease of the cortical neurons in the cerebrum. Emil Kraepelin named this condition of serious concern as Alzheimer’s disease (AD), taken after the name of Alois Alzheimer (DeTure and Dickson, 2019; Breijyeh and Karaman, 2020). Advanced reduction in cognition can be brought about by cerebro-cortical disorder like AD (Kelley and Petersen, 2007). Other suggested causes are boozing, microorganism infections, anomaly of cardiovascular and/or pulmonary systems that can reduce oxygen supply to the brain, nutritional deficit, cyanocobalamin deficiency and cancers (Baazaoui and Iqbal, 2022; Holtzman et al., 2011; Rathod et al., 2016). Currently, about 50 million people are affected globally by AD. The numbers are expected to double every 5 years and to almost triple by 2050 (Livingston et al., 2020; Nichols et al., 2022). AD overburden the affected individuals, their families and countries’ economies. In fact, the total health expenditure of AD alone as per researchers is over US$1 trillion per annum (Chen et al., 2024). Until now, AD is incurable. The existing pharmacological interventions can only manage AD’s signs and symptoms (Passeri et al., 2022). Researchers classified AD as multifactorial disease; two main etiologies were hypothesised: the acetyl cholinergic and amyloid beta (Singh et al., 2024). At present, drugs which are approved to treat AD are acetylcholinesterase inhibitors and *N*-methyl d-aspartate (NMDA) antagonists (Barthold et al., 2020). Globally, researchers are trying to explore the mechanism involved behind the progressive neurodegeneration of cerebral cholinergic neurons, abnormal tau protein metabolism, beta-amyloid, inflammation and oxidative free radical damage. The aim is to develop promising pharmacological entities that can stop or modify the neurodegeneration in AD (Forsyth and Ritzline, 1998)

Oseltamivir is an antiviral, which acts by inhibiting viral neuraminidase that is required for the virus to be released from the host cells. Researchers reported that oseltamivir reduces inflammation. It is one of the mechanisms suggested in the reduction of influenza symptoms (Bird et al., 2015) and in the production of antipyretic effect (Treanor et al., 2000). Oseltamivir is primarily used for the treatment of influenza A and B infections. Oseltamivir’s target viral enzyme neuraminidase cleaves the sialic acid residues present terminally on carbohydrate moieties of host cell membrane and influenza virus envelop (Treanor et al., 2000). This process breaks down the membrane barriers and causes the release of progeny viruses from the host cells. Through the same process, new virion infects other cells. The neuraminidase inhibitors bring about the static infectious condition and make available these virions to the macrophages intracellularly (McKimm-Breschkin, 2013).

Sialidases are recognised as analogues to viral neuraminidase in humans, and they have been termed as human neuraminidases (hNEU). Exploration of hNEU chemistry and pharmacology revealed to exhibit the same structure and function as that of viral neuraminidase (Magesh et al., 2009; Glanz et al., 2018; Keil et al., 2022). Four isomers of sialidase, which are now known as hNEU, have been recognised, and they hydrolyse sialosides, gangliosides and glycoproteins in humans (Seo et al., 2021). These enzymes were found to play pivotal roles in different pathological states such as diabetes, (Easwaran et al., 2023a), cancers (Reddy et al., 2015) and neurodegenerative disorders (Miyagi and Taniguchi, 2008). hNEU-1 is the most abundant isoform of enzyme among all hNEU, which is present in lysosomes and plasma membrane and is a part of membrane complex (Maurice et al., 2016). hNEU-1 cleaves sialic acid from oligosaccharides and glycoproteins, and it has no effect on gangliosides. Sialidase (hNEU-2) localises in cytosol and catalyses sialic acid from verities of glycans. Sialidase (hNEU-3) is predominantly associated with plasma membrane and it selectively causes desialylation of gangliosides. Fourth isoform of sialidase (hNEU-4) exists mainly on membranes of cell organelles and possesses broad choice of glycan selectivity (Magesh et al., 2006).

Sialidases mediate cleavage reaction of glycoproteins, oligosaccharides and gangliosides. Sialidases also release sialic acid, which has been found to be involved in verities of biological processes including cancers, diabetes, neurodegeneration, inflammation and many more (Seo et al., 2021). Sialic acid releases play homeostatic as well as pathological roles. Sialic acid’s influence on neurons has been extensively studied by the researchers, and it has been found that sialidase mutations and generations of sialic acid are negatively associated with AD (Zhu et al., 2024). Sialic acid is required to activate many Siglec genes (primarily downregulated inflammation), which are required in stressful microglial environment and to protect the microglial cells (Khatua et al., 2013). However, this balance of microglial activation and inflammation disrupts in chronic cases and becomes the basis of neurodegeneration due to inflammatory upregulation (Gao et al., 2023). Sialic acid on gangliosides interacts with Ca^+2^ and facilitates the synaptic transmission, which in excess can lead to excitoxicity (Chahinian et al., 2024). Studies with increased serum sialic acid concentration in AD exhibited sialic to mediate the amyloid β (Aβ) association with gangliosides, which in turn leads to the Aβ plaque formation (Xiao et al., 2022). Sialic acid has been found to downregulate Siglec-3 (CD33). The stimulation of Siglec-3 is very well established in microglial protection (Puigdellívol et al., 2020).

By reviewing the abovementioned literature and other related studies about the sialidase (hNEU), we came to hypothesise that hNEU can be a good target to inhibit the neurodegeneration from multiple fronts which are opened because of neuraminidase stimulation and liberation of sialic acid. Considering the fact that oseltamivir is a viral neuraminidase (vNEU) inhibitor and sialidases in humans are the structural analogues to (vNEU), we aimed to study the impact of neuraminidase inhibitor, i.e., oseltamivir against neuraminidase and different analogues of human neuraminidase including secretases, glycogen synthase kinase, cyclin dependent kinase, protein phosphatase 2A, sialyltransferase, acetylcholinesterase, interleukin-1B and tumour necrosis factor through preliminary pharmacological and toxicity profiling, molecular docking and molecular dynamic simulations. We aim to understand the role of different genes through gene expression analysis to establish the strength of interaction of oseltamivir against various enzymes mentioned and to shed light on its behaviour resembling cellular nature and its expression in the influence of different genes which are pivotal in human biology.

## Methods

### Preparing the protein for insilico processing

Proteins of interest, which play a significant role in the dysregulated metabolism, were identified as follows: neuraminidase (PDB ID: 2HTY), responsible for the entry of viral particles inside the healthy mammalian cells; (McAuley et al., 2019); beta secretase (PDB ID: 5YGX), responsible for the proteolytic cleavage of AD amyloid precursor; (Sambamurti et al., 2007); gamma secretase (PDB ID: 6YHF), responsible for the breakdown of the amyloid precursor protein to amyloid beta; (Carroll and Li, 2016); alpha secretase (PDB ID: 6BE6), responsible for the breakdown of amyloid precursor protein to soluble amyloid precursor protein alpha; (Corbo et al., 2011); glycogen synthase kinase – 3 beta (GSK3B) (PDB ID: 1PYX), responsible for the regulation of signalling post endocytic transport; (Partonen and Kushida, 2023); cyclin dependent kinase – 5 (CDK5) (PDB ID: 1H4L), responsible for organising the cytoskeleton and cellular growth; (Tian et al., 2022); protein phosphatase 2A (PP2A) (PDB ID: 1B3U), responsible for the cellular processes like autophagy, apoptosis, cell proliferation and DNA repair; (Dzulko et al., 2020); sialyltransferase (PDB ID: 5BO6), responsible for the hydrolysis of sialic acid; (Zhang et al., 2010); acetylcholinesterase (PDB ID: 1ACJ), responsible for the metabolism of acetylcholine at the synapse of two different neurons; (Volkow et al., 2001); interleukin 1B (PDB ID: 1IOB), responsible for the endocrine and reproductive dysfunction; (Baraskar et al., 2021); and, tumour necrosis factor (PDB ID: 1TNF), responsible for the modulation of various gene expression cellularly. (Schütze et al., 1992). The protein pdb files were downloaded from the RCSB PDB database (https://www.rcsb.org/) and (Berman et al., 2000) visualised through BIOVIA discovery studio (Baroroh et al., 2023) application, where the hetatms, water and co-crystals were removed and polar hydrogens were added to induce the charge around the protein structure to facilitate perfect interaction with the ligand in case of molecular docking and MD simulations.

### Preparing oseltamivir as ligand

The structure of oseltamivir was downloaded in the .sdf format, which will be formatted as ligand of the study. Furthermore, it was downloaded from structural information rich databases like ‘PubChem’ directory (https://pubchem.ncbi.nlm.nih.gov/), (Kim et al., 2024) particularly oseltamivir (ethyl (3*R*,4*R*,5*S*)-4-acetamido-5-amino-3-pentan-3-yloxycyclohexene-1-carboxylate) (Green et al., 2008). The processing of the molecule for the conversion into the pdbqt was done by employing open babel tool (O'Boyle et al., 2011).

### Identification of pharmacological and toxicological activity employing PASS tool

The prediction of activity spectra of substances (PASS) (https://www.way2drug.com/passonline/), an online tool, was selected to predict the possible activity of oseltamivir. The molecular SMILES formula was given as input in the interface of PASS, which predicts the possible pharmacological actions and toxicological activities. The structure-activity relationship was denoted by Pa: probability of activity and Pi: probability of inactivity. This approach provided an idea concerning the possible targets and activity for approaching through preparing the biomolecules of interest (enzymes) for further processing, employing molecular docking and MD simulation studies to develop an understanding with the evidence (Poroikov et al., 2007).

### Performing the proteins - ligand molecular docking

The processed protein crystal structures were uploaded into the PyRx interface (https://pyrx.sourceforge.io/) and converted into pdbqt format as a macromolecule of the study (Dallakyan et al., 2015). The ligands.sdf file was uploaded through the ‘Open Babel’ and was processed into pdbqt format to make ligand molecule for molecular docking after energy minimisation. The molecular docking was performed by creating a grid dimension (Table 2), and selecting complete protein. Twenty core CPUs with effectiveness were selected, and final docked molecules were saved for further processing. BIOVIA and discovery studios were the preferred methods to analyse the results obtained after molecular docking, including the 2D structures of proteins and ligands interactions, protein-ligand interactions and pocket identification (Murugan et al., 2022; Germoush et al., 2024).

### Preparing the enzyme and oseltamivir for MD simulation

Pymol interface was selected for converting the.dsv files into.pdb files. The separately imported protein and ligand files after molecular docking were joined through pymol. It was then saved in the form of pdb file for performing the MD simulation by means of GROMACS for a time period of 50 nanoseconds (ns), temperature of 300K, solvent–water, ions–sodium and chlorine, with one random seed and selected seed of seed_1234 for duality (Bytheway et al., 2008; Coutsias and Wester, 2019; Sasumana and Kaushik, 2018; Varghese et al., 2024; Yuan et al., 2017; Bekker et al., 1993).

The molecular dynamic (MD) simulation of a oseltamivir-neuraminidase complex was performed using GROMACS 2024, installed on linux (ubuntu) operating system, supported by the NIVIDIA RTX 4060 graphical processing unit and 16 GB RAM. Firstly, the topology files of the protein was generated using GROMACS compatible forcefield such as CHARMM36 and the ligand topology files were generated using Swissparam tools (Bugnon et al., 2023; Zoete et al., 2011; Yesselman et al., 2012). Secondly, the ligand-protein complex was assembled and a simulation box was defined maintaining a minimum distance of 1.0 nm from the edges. TIP3P water model was chosen to solvate the simulation box was used. Sodium and chlorine was used as counterions to neutralise the system. To reduce steric clashes, energy minimising of the system was performed employing the steepest descent algorithm, followed by two equilibration steps such as NVT and NPT for a time period of 100 ps (picoseconds) to stabilise the temperature and pressure of the system using the V-rescale and Parrinello–Rahman algorithms. The production MD simulation run was conducted for 50 ns (nanoseconds) with a time step of 2 fs under stable boundary conditions saving the results every 10 ps.

Build in GROMACS utilities were used to perform post simulation analysis. The structural stability of the complex was assessed by calculating the root mean square deviation (RMSD) of the ligand and protein over time. While flexibility was evaluated using root mean square fluctuation (RMSF) per residue and the radius of gyration was assessed to determine the compactness of the protein. Binding free energies were carried employing the gmx_MMPBSA tool, externally installed using anaconda3 packages. The MD trajectory and topology files were used for processing gmx_MMPBSA (Molecular Mechanics Generalized Born Surface Area) and gmx_MMGBSA (Molecular Mechanics Poisson–Boltzmann Surface Area) binding free energies over represented snapshots. (Miller et al., 2012). The energies included van der Waals, electrostatic, polar solvation and non-polar solvation contributions. This protocol provided insights into the stability, dynamics, and binding energetics of the ligand–protein complex.

### Performing the gene expression analysis of sialyltransferase (analogue of neuraminidase)

Gene expression analysis was executed by means of GEO2R (https://www.ncbi.nlm.nih.gov/geo/geo2r/), an online tool of national centre for biotechnological information (NCBI) database (https://www.ncbi.nlm.nih.gov/) for variance expression analysis of huge datasets from gene expression omnibus (GEO). Datasets were normalised and comparisons between case and control groups were achieved. Significant genes were identified based on p-values (<0.05) and log 2-fold change thresholds for the gene analysis (Kalyan et al., 2023). The GEO series accessions involving sialyltransferase genes expression data, i.e., GSE5281 (Liang et al., 2007; Liang et al., 2008a; Readhead et al., 2018; Liang et al., 2008b), were selected for the study. The gene expression data was examined using dataset GSE5281, bearing the genes responsible for the development of AD and bearing the data of 61 control samples and 44 case samples. Differential expressional analysis was performed to recognise significant gene regulated within the groups. The study was completed using Benjamini and Hochberg method with NCBI generated data following the statistically significant level cut-off of p-values (<0.05), log 2-fold change threshold ‘zero’ with volcano and mean difference plot for control group vs. case group. The results are expressed in Table 4, bearing the data of twenty highly significant genes associated with the development or that played a crucial role in the pathway related to AD.

### Gene enriching analysis

To identify the biological impact of twenty significant genes derived out of GEO analysis associated with AD, the gene enrichment analysis was performed employing the enrichr tool. The significant gene codes were given as input into the enrichr interface, (Kuleshov et al., 2016; Chen et al., 2013), which integrates the data from various databases. The analysis involves selecting relevant categories like gene ontology (GO), (Ashburner et al., 2000), terms for cellular components, molecular functions and biological processes. Furthermore, disease specific annotations were explored employing Jensen diseases and Jensen tissue databases to understand the link of the genes with the AD pathology. Adjusted p-values (Padj) were used to ensure statistical significance. Combined scores, which integrate p-values and z-scores, were used to rank the enriched outcomes so that the results provide insights into the role of synaptic function, protein phosphorylation, lipid metabolism and neuroinflammation, which are critical in understanding the pathology of AD (Xie et al., 2021). The findings were visualised using volcano plots to understand and recognise key pathways and phenotypes for further investigation.

## Results

The PASS prediction analysis points out several important pharmacological activities with high probable activities (Pa) and low possibility of inactivity (Pi), which suggests the oseltamivir is highly active against the targets like Neuraminidase inhibitor (Pa = 0.931) and Alpha-N-acetylglucosaminidase inhibitor (Pa = 0.455). The aforementioned targets could be of prime interest as the enzyme may be involved in the transferase reaction and may result in the production of abnormal proteins. Three different variants were identified based on the mutated gene expression, which could result in the development of neuronal disorders (Alroy et al., 2014). Inhibition of enzymes, highlighting its role in neuronal protection, misrepresentation or misexpression of the gene related to this enzyme, may result in neurological disorders, which is the point of interest of the current study Deficiency of the said enzyme may result in various other neurological disorders (Zhao and Neufeld, 2000). Additionally, it may result in toxicities like metabolic acidosis (0.511), sneezing, twitching, weight loss, hypocalcemia, dyspnea, gastrointestinal disturbance, ototoxicity, sensitization and thrombophlebitis with lower intensity, which are needed to be taken care of during the evaluation of the study. These activities express oseltamivir’s resourcefulness and therapeutic potential in diverse biological contexts related to Alzheimer’s disease (Table 1). (Annexure 1).

**TABLE 1:** The activity prediction of oseltamivir.

Pharmacological activity prediction		
Pa	Pi	Predicted activity
0.931	0.000	Neuraminidase (Influenza B) inhibitor
0.932	0.001	Antiviral (Influenza)
0.920	0.001	Antiviral (Influenza A)
0.685	0.000	Neuraminidase (influenza) inhibitor
0.652	0.005	Macrophage stimulant
0.455	0.013	Alpha-N-acetylglucosaminidase inhibitor
0.473	0.048	Muramoyltetrapeptide carboxypeptidase inhibitor
0.461	0.042	Antiviral (Rhinovirus)
0.447	0.034	Peptide-N4-(N-acetyl-beta-glucosaminyl) asparagine amidase inhibitor
0.434	0.034	Antimyopathies
Toxicity Prediction		
Pa	Pi	Predicted activity
0.511	0.063	Acidosis, metabolic
0.463	0.082	Sneezing
0.517	0.190	Twitching
0.417	0.110	Weight loss
0.359	0.065	Hypocalcaemic
0.375	0.097	Dyspnea
0.333	0.127	Gastrointestinal disturbance
0.326	0.129	Ototoxicity
0.259	0.084	Sensitization
0.363	0.195	Thrombophlebitis

Pa: probably activity, Pi: possibly inactivity, data derived through an online tool, i.e., PASS program (http://www.way2drug.com/PASSOnline/).

### Molecular docking

**FIGURE 1:**
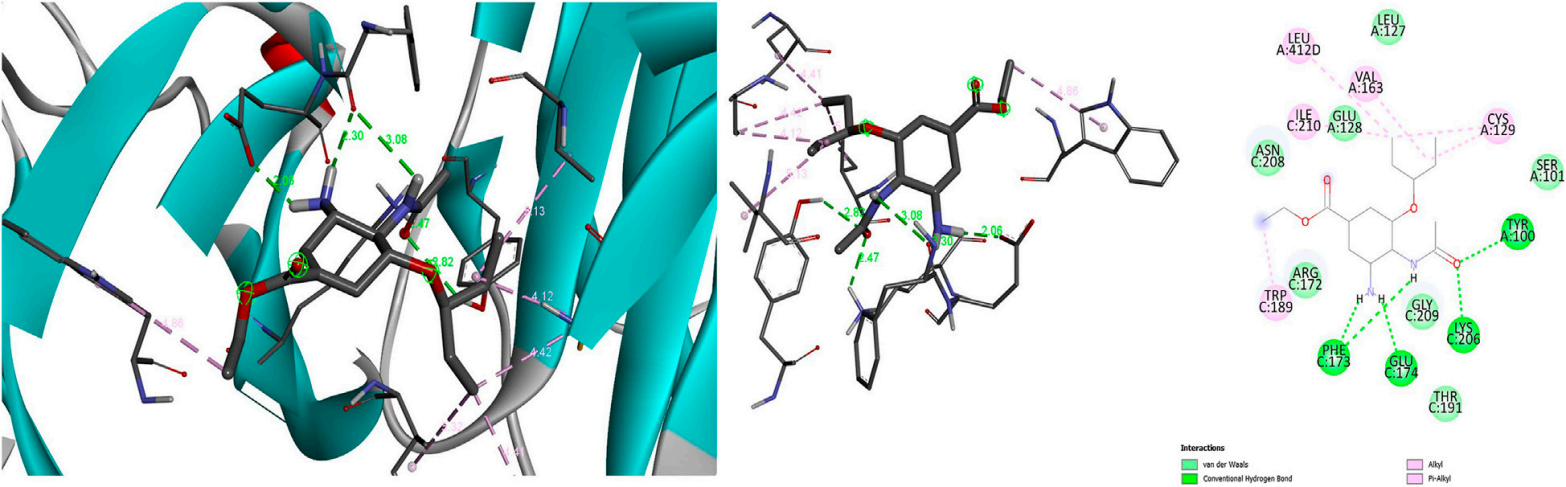
shows the interaction between the ligand (oseltamivir) and the enzyme protein (neuraminidase); first from left shows the receptor interaction with ligand, middle image shows the protein interactive pocket atoms with ligand and the third image shows the 2D illustration of amino acids interacting with ligand through hydrogen, Vander Waal’s, among other interactions.

The oseltamivir interacted with neuraminidase at a hydrogen bond length of 2.06Å with GLU C:174, expressing the lowest binding score of −6.5 kcal/mol at given grid values mentioned in Table 2. Further, the interaction between protein and ligand was formed even through Vander wall’s interaction with ARG C:172 along with six other amino acids. The oseltamivir also interacted with beta secretase at a hydrogen bond length of 1.86Å with TYR A:71, expressing the lowest binding score of −6.2 kcal/mol. Further, the interaction between protein and ligand was formed even through Vander wall’s interaction with ILE 118 along with nine other amino acids. The oseltamivir interacted with gamma secretase as well at a hydrogen bond length of 3.02Å with ALA 42, expressing the lowest binding score of −3.5 kcal/mol, a bit weaker interaction in comparison with other proteins. Further, the interaction between protein and ligand was formed even through Vander wall’s interaction with ILE 45 along with three other amino acids. Similarly, the oseltamivir interacted with alpha secretase at a hydrogen bond length of 2.14Å with ARG A:239, expressing the lowest binding score of - 6.4 kcal/mol. Further, the interaction between protein and ligand was formed even through Vander wall’s interaction with THR A:238 along with eight other amino acids. The oseltamivir interacted with glycogen synthase kinase 3 beta (GSK-3B) too at a hydrogen bond length of 2.05Å with GLY B:202, expressing the lowest binding score of - 6.6 kcal/mol. Further, the interaction between protein and ligand was formed even through Vander wall’s interaction with LYS A:292 along with six other amino acids. Additionally, the oseltamivir interacted with cyclin dependant kinase 5 (CDK-5) at a hydrogen bond length of 2.87Å with ASP A:86, expressing the lowest binding score of - 6.3 kcal/mol. Further, the interaction between protein and ligand was formed even through Vander wall’s interaction with 10 other amino acids. There was also an interaction of oseltamivir with protein phosphatase 2A (PP2A) at a hydrogen bond length of 1.94Å with ARG A:104, expressing the lowest binding score of - 5.5 kcal/mol. Further, the interaction between protein and ligand was formed even through Vander wall’s interaction with 12 other amino acids. Correspondingly, the oseltamivir interacted with sialyltransferase at a hydrogen bond length of 2.12Å with THR B:194, expressing the lowest binding score of - 6.4 kcal/mol. Further, the interaction between protein and ligand was formed even through Vander wall’s interaction with six other amino acids. In the same manner, the oseltamivir interacted with acetylcholinesterase at a hydrogen bond length of 2.55Å with TRP A:84, expressing the lowest binding score of - 7.9 kcal/mol, which is the highest binding affinity of the current study. Further, the interaction between protein and ligand was formed even through Vander wall’s interaction with 19 other amino acids. Adding to the list, the oseltamivir interacted also with interleukin 1B at a hydrogen bond length of 2.02Å with LEU A:62, expressing the lowest binding score of - 5.3 kcal/mol. Further, the interaction between protein and ligand was formed even through Vander wall’s interaction with nine other amino acids. Lastly, the oseltamivir interacted with tumour necrosis factor - alpha at a hydrogen bond length of 2.37Å with SER C:99, expressing the lowest binding score of - 7.4 kcal/mol, which is also one of the best results of the current study. Further, the interaction between protein and ligand was formed even through Vander wall’s interaction with 14 other amino acids. (Table 2) (Figure 1).

**TABLE 2:** Protein (Neuraminidase) -Ligand (Oseltamivir) Docking Results of ‘*PyRx Python Prescription 0.8*’ Analysed Through ‘*BIOVIA-Discovery Studios Visualiser v.21.1.0.20298*’.

Name of ligand (Oseltamivir)	Name of protein	Various receptor enzymes associated with Alzheimer’s as target proteins and oseltamivir as ligands						
		*Highest affinity score* *Kcal/mol*	*RMSD*	*Ionic or wander wall’s interactions (Å units)*	*Hydrogen bond interactions (Å units)*	*Amino acids*	*Grid dimensions* *Centre Dimensions*	
Oseltamivir 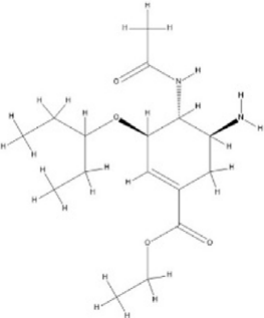	Neuraminidase (PDB ID: 2HTY) 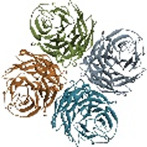	−6.5	0	ARG C:172, LEU A:127, SER A:101, ASN C:208, GLU C:209, GLU A:128, THR C:191	2.30 2.06 2.47 2.82	PHE C:173, GLU C:174, LYS C:206, TYR A:100	x = 1.5994 y = 50.1814 z = 100.559	x = 103.0816 y = 102.4211 z = 53.8510
	Beta secretase (PDB ID: 5YGX) 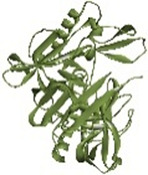	−6.2	0	ILE 118, ARG 128, SER36, GLY 34, TYR 198, ILE 226, ASP 228, VAL 332, ASP 32, THR 231	1.86 2.15	TYR A:71 GLY A:230	x = 14.3015 y = 41.0494 z = 0.1888	x = 56.1419 y = 64.6465 z = 45.9909
	Gamma secretase (PDB ID: 6YHF) 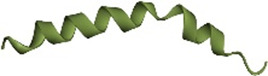	−3.5	0	ILE 45, GLY 38, LEU 34, MET 35	3.02	ALA 42	x = - 2.2973 y = −3.2008 z = 11.7049	x = 26.2145 y = 48.5674 z = 13.6442
	Alpha secretase (PDB ID: 6BE6) 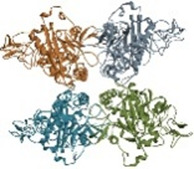	−6.4	0	THR A:238, LYS A:518, GLU A:240, ILE A:243, SER A:506, SER A:504, CYS A:473, ASP A:481, LYS A:480	2.14	ARG A:239	x = 31.7668 y = 33.1965 z = 18.0663	x = 94.5299 y = 109.4674 z = 123.8035
	GSK-3B (PDB ID: 1PYX) 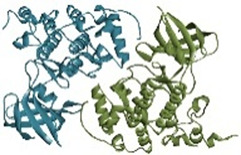	−6.6	0	LYS A:292, ASN B:95, GLN B:89, VAL B:87, LYS B:85, SER B:203, PHE B:67	2.51 2.57 2.05	ARG B:96 GLU B:97 GLY B:202	x = 24.2110 y = −0.3042 z = 21.4266	x = 72.9735 y = 80.3676 z = 103.6807
	CDK-5 (PDB ID: 1H4L) 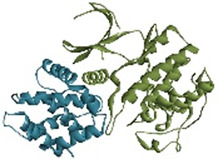	−6.3	0	GLU A:81, ALA A:143, ASP A:84, GLN A:85, ILE A:10, GLN A:130, ASN A:131, ASP A:144, LYS A:33, VAL A:18	2.87	ASP A:86	x = 35.4578 y = −32.768 z = 26.1504	x = 77.4488 y = 60.5521 z = 49.0611
	PP2A (PDB ID: 1B3U) 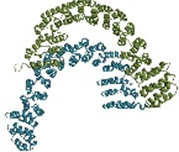	−5.5	0	THR A:141, ALA A:183, SER A:186, LYS A:187, GLY A:148, CYS A:143, VAL A:108, SER A:145, THR A:101, ARG A:182, MET A:179, PHE A:140	2.54 2.37 1.94	ASP A:105 THR A:144 ARG A:104	x = 34.6630 y = 46.7673 z = 23.0447	x = 97.3458 y = 107.1759 z = 122.2308
	Sialyltransferase (PDB ID: 5BO6) 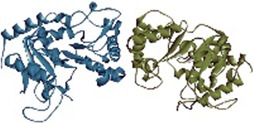	−6.4	0	ALA B:192, LEU B:238, GLU B:195, SER B:234, ASN B; 230, PRO B:190	2.12	THR B:194	x = −3.5210 y = 9.8904 z = −11.0690	x = 61.7766 y = 59.4714 z = 102.3049
	Acetylcholine-esterase (PDB ID: 1ACJ) 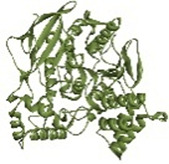	−7.9	0	GLU A: 199, GLY A:441, TYR A:130, HIS A:440, GLY A:117, TYR A:442, LEU A:127, SER A:124, GLY A:118, GLY A:123, SER A:122, ASN A:85, GLN A:69, TYR A:121, ASP A:72, GLY A:80, TRP A:432, SER A:81, TYR A:334	2.55	TRP A:84	x = 4.7576 y = 65.5208 z = 56.8290	x = 64.6127 y = 61.3047 z = 56.9408
	Interleukin 1B (PDB ID: 1IOB) 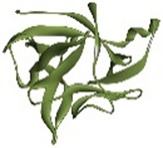	−5.3	0	VAL A:85, ASN A:66, TYR A:90, ASN A:7, SER A:5, SER A:43, GLY A:61, GLU A:64, LYS A:65	2.02 2.39	LEU A:62 LYS A:63	x = 15.9224 y = 13.5665 z = 1.1895	x = 44.2915 y = 37.7782 z = 44.89.24
	TNF alpha (PDB ID: 1TNF) 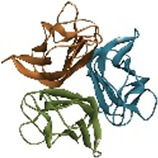	−7.4	0	LYS C:98, PRO B:110, LYS A:98, GLU C:116, GLU B:116, SER B:99, GLU A:116, PRO A:100, TYR A:115, TRP A:114, CYS A:110, PRO C:100, GLN C:102, GLU C:104	2.37 3.02	SER C:99 GLN B:102	x = 19.9745 y = 49.6645 z = 39.9343	x = 62.7877 y = 63.9782 z = 61.4350

The summary of the dual MD simulation (random simulation and seed_1234) of the oseltamivir-neuraminidase complex is explained in Table 4, which consist a of binding free energies components as integral part of MM-PBSA analysis. Significant high magnitude of van der Waals interactions (ΔE_vdW) changed form the negligible phase in pre-dynamic MM-PBSA (−0.00 ± 1.26 for random and 0.00 ± 0.40 for seed_1234) to a considerable negative value post-simulation (−20.22 ± 0.73 and −19.05 ± 0.50), which indicates high binding confirmation and attractive ness between the ligand and protein. Similar observations were noticed for electrostatic interactions (ΔE_elec), the energy quotient shifts from negligible to highly promising post-dynamic i.e., −284.37 ± 0.95 for random and −255.35 ± 1.46 for seed_1234, these results confirm the strong bonding between the ligand and protein. However, in case of polar solvation energy (ΔG_polar) the post simulation results of random seed was increased and seed_1234 was decreased, showing a system determined correction was pointed to the seed_1234 simulation being more favourable. The total gas-phase energy (ΔG_gas), transforms in to significantly favourable value post simulation, indicating stronger intermolecular connections in the complex environment. Finally, the binding free energies (ΔG_bind) change from the positive in pre-MM-PBSA value to negative in post MM-PBSA value exhibits improved binding characteristics post molecular dynamics.

The results MM-GBSA analysis of pre and post dual MD simulation of random seed and seed_1234 for determining their binding free energies is expressed in Table 5, the van der Waals interaction energy (ΔE_vdW) demonstrated shift from undermined values in pre dynamic simulation (0.00 ± 0.00 random and −0.00 ± 0.00 seed_1234) to recognisable post dynamic simulation (−18.78 ± 0.01 for random and −19.18 ± 0.78 for seed_1234) values representing better packing between ligand and protein post simulation. Electrostatic interactions (ΔE_elec) showed similar results, shifting from insignificant range of values to desirable values (−266.20 ± 0.46 for random and −259.86 ± 1.81 for seed_1234) post simulation. Conclusively, the total binding free energies (ΔG_bind) remained constantly negative in both pre and post MM-GBSA states, suggesting the stable and promising binding between the ligand and protein.

The MM-GBSA decomposition analysis for the ligand and protein complex, pre and post MD simulations in random and fixed (seed_1234) conditions revealed that key amino acid fragments such as GLU:276 and GLU:277 developed strong interactions in pre-MD simulation analysis with values −10.36 and −11.98 kcal/mol under random conditions. A great shift in the energies of the amino acids was identified in post MD simulation, i.e., GLU:276 reaching −15.61 kcal/mol and GLU:277 displaying a positive value of 3.87 kcal/mol, highlighting important changes. Residues like ARG:224 and ARG:292 better binding energies post MD simulations and LEU:134, TRP:178, SER:179, and ILE:222 maintained constant negative energies throughout simulations.

**FIGURE 2:**
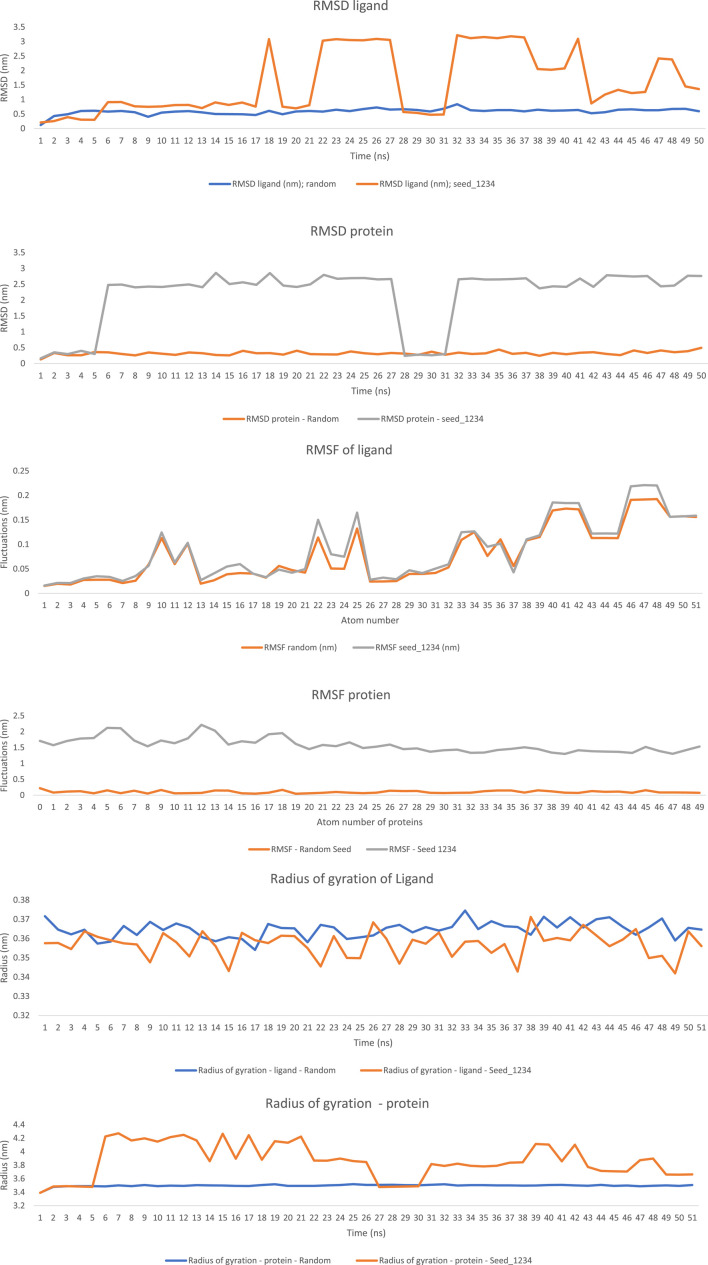
Represents the molecular dynamic simulation interactions of the ligand and protein complex as RMSD of protein and ligand, RMSF of protein and ligand, radius of gyration.

The interaction of ligand, oseltamivir, and enzyme protein, neuraminidase, was analysed through molecular dynamic simulation employing GROMACS tool. Various crucial parameters have been identified such as RMSD, radius of gyration (Rg), Vander Waal’s surface area (VSA) and the potential energy of the complex (PE). The protein RMSD ranged for Protein: P: 0.128424–0.493372 nm, ligand 0.122013–0.838708 nm under random seed simulation and under seed_1234 simulation it ranged from Protein 0.161276–2.858013 nm and Ligand 0.208401–3.217241 nm, which explains that the ligand-protein complex has undergone minimal structural disorientation and exhibited stable binding throughout the test. The radius of gyration remained constant between for protein at a range of 3.391135–3.518412 nm and for the ligand at a range of 0.354091–0.37452 nm under random simulation conditions, however, the protein and ligand showed the radius of gyration of about 3.390434–4.271447 nm and 0.341893–0.371202 nm under seed_1234 simulation conditions which explains minimal disorientation in seed_1234 simulation. The RMSF of protein and ligand under random and seed_1234 MD simulation conditions exhibited a value of P: 0.0451–0.2189 nm, L: 0.0153–0.1925 nm for random simulation and P: 1.2986–2.2136 nm and ligand L: 0.0160–0.2210 nm under seed_1234 simulation conditions exhibiting vigorous molecular interactions between the ligand and protein in a complex, which justify the results expressed in Tables 2, 3. Through this current simulation results, it is evident that the oseltamivir forms a stable and favourable bond with the enzyme protein like neuraminidase, which underscores the oseltamivir as a suitable candidate for development of pharmacophore against the enzyme protein of neuraminidase (Figure 2).

**TABLE 3:** Molecular dynamic (MD) simulation of oseltamivir and neuraminidase.

*Seed*	*Protein*	*Ligand*	*Root mean square deviation (RMSD) nm*	*Root mean square fluctuations (RMSF) nm*	*Radius of gyration (Rg) nm*
Random seed	Neuraminidase	Oseltamivir	P: 0.128424–0.493372 L: 0.122013–0.838708	P: 0.0451–0.2189 L: 0.0153–0.1925	P: 3.391135–3.518412 L: 0.354091–0.37452
Seed_1234	Neuraminidase	Oseltamivir	P: 0.161276–2.858013 L: 0.208401–3.217241	P: 1.2986–2.2136 L: 0.0160–0.2210	P: 3.390434–4.271447 L: 0.341893–0.371202

nm = Nanometres, P = protein, L = ligand.

Through these results, it can be explained that targeting the neuraminidase can ensure effective modulation of pathological pathway related to AD (Du et al., 2024), as neuraminidase or similar enzymes like sialic acid modifying agents are involved in neurodegenerative pathways (Zhang et al., 2023).

**FIGURE 3:**
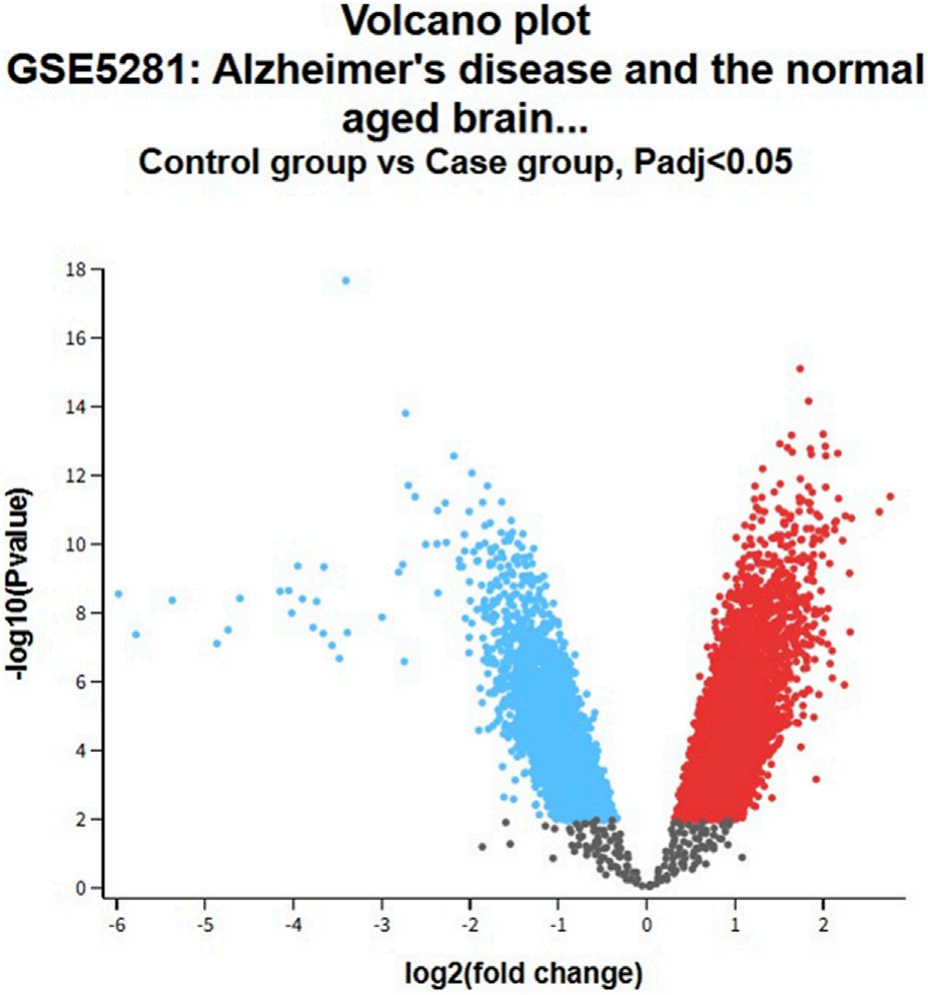
Volcano plot of gene expression related to sialyltransferase; red colour shows the upregulation of genes and the blue colour shows the downregulation of genes.

The dataset validation performed through GEO employing the accession number GSE5281 and focusing on the gene expression related to the Alzheimer’s disease for sialyltransferase, which is an analogue of neuraminidase in humans, has shown significant differential expression results. Based on their low p-value and high log fold-change (Log FC) ranging between 6.25 × 10^−10^ to 3.92 × 10^−8^, significant statistical evidence for changes related to gene expression was found. The log FC values of 1 shows two folds, 1.5 three folds and 2 four folds in the gene upregulation in the given dataset. The significantly expressed genes like tubulin beta 3 class III (TUBB3) and synaptosome associated protein 25 (SNAP25) are important for normal neuronal structure and function even at the synaptic level (Duly et al., 2022; Olsson et al., 2011), the processes which are dysregulated in AD. The four folds upregulation of ubiquitin C-terminal hydrolase L1 (UCHL1) and PPP2R2D, a subunit of protein phosphatase 2A, suggests altered signalling pathways and protein turnover in human brain (Bishop et al., 2016), which are associated with neurodegenerative diseases. The neurexin 3 (NRXN3) and calmodulin 1 (CALM1) are associated with synaptic connectivity, calcium signalling and disrupting, which are highly associated with AD. (Mu et al., 2024; Chin and Means, 2000) Other genes like fatty acid binding protein 3 (FABP3), (Lee et al., 2020), which represents the lipid metabolism, and somatostatin (SST) (Samson et al., 2008) have neuromodulatory function highly affecting the cognitive processes. Dysregulation of which may develop the AD. (Samson et al., 2008) Aggregation of certain protein and inflammation of developing genes such as reticulon 3 (RTN3) (Grumati et al., 2017) and C1q and tumour necrosis factor-related protein 4 (C1QTNF4) (Vester et al., 2021) can act as central to the development and prognosis of AD. Through this analysis, the network of dysregulated genes involved in synaptic function, neuroinflammation, calcium function and protein homeostasis can be explained. The significant alteration in the gene expression and homeostasis emphasises potential targets for novel therapeutic intervention in relation to AD. (Table 4) The volcano plot expresses the differential expressing gene between Alzheimer’s disease samples and normal samples obtained from the dataset GSE5281 (Figure 3), which shows distinct upregulated and downregulated genes in the context of AD. In the log FC values, positive values (red) show upregulated genes while negative values (blue) show downregulated genes. The y-axis (p-value) shows the significance of the study samples, where the non-significant genes are shown in grey colour. These genes may be involved in inflammation, synaptic dysfunction, amyloid metabolism or impaired neuroprotection. These findings provide potential biomarkers for the development of novel therapeutic agents. (Annexure II).

**TABLE 4:** Results of pre and post MM-PBSA binding free energy components in kcal/mol of the MD simulated molecular complex of Oseltamivir and Neuraminidase.

Energy components	Pre MM-PBSA (Mean ± SD); random seed	Post MM-PBSA (Mean ± SD); random seed	Pre MM-PBSA (Mean ± SD); Seed_1234	Post MM-PBSA (Mean ± SD); Seed_1234
ΔE_vdW (van der Waals)	−0.00 ± 1.26	−20.22 ± 0.73	0.00 ± 0.40	−19.05 ± 0.50
ΔE_elec (Electrostatic)	−0.00 ± 2.22	−284.37 ± 0.95	0.00 ± 3.20	−255.35 ± 1.46
ΔG_polar (Polar solvation)	246.03 ± 17.02	289.19 ± 1.33	267.15 ± 11.36	263.47 ± 0.31
ΔG_nonpolar (Non-polar solvation)	−3.56 ± 0.03	−3.25 ± 0.04	−3.57 ± 0.03	−3.31 ± 0.05
ΔG_gas (Energy in vacuum)	−229.62 ± 20.44	−304.59 ± 1.20	−269.62 ± 6.13	−274.40 ± 1.56
ΔG_solvation (Total solvation energy)	242.47 ± 17.02	285.95 ± 1.33	263.58 ± 11.36	260.16 ± 0.31
ΔG_bind (Total Binding Free Energy)	12.84 ± 26.60	−18.65 ± 1.79	−6.04 ± 12.91	−14.24 ± 1.59

ΔE_vdW: van der Waals interaction energy between the ligand and the protein.

ΔE_elec: electrostatic interaction energy.

ΔG_polar: solvation free energy due to polar interactions, calculated using the Poisson–Boltzmann (PB) or Generalized Born (GB) models.

ΔG_nonpolar: non-polar solvation free energy, often estimated from the solvent-accessible surface area (SASA).

ΔG_gas: Total energy in vacuum (ΔE_vdW + ΔEEL).

ΔG_solvation: Total solvation energy (ΔG_polar + ΔG_nonpolar).

ΔG_bind: total binding free energy, sum of the above components.

The enricher-based analysis of 20 highly significant genes has revealed an enriching analysis of genes implicated in Alzheimer’s disease, highlighting significant association among the cellular components, molecular function, mammalian phenotypes, tissues and related diseases. Within the cellular components, vesicles (adjusted p-value = 0.04964, combined score = 115.27) and glutamatergic synapses (adjusted p-value = 0.04964, combined score = 200.25) emerged as critical, emphasising the known roles of synaptic dysfunction in AD. In molecular function, kinase binding (adjusted p-value = 0.03205, combined score = 89.70) and protein phosphatase regulator activity (adjusted p-value = 0.03324, combined score = 278.70) highlighted the disruption in protein phosphorylation as a tau-related AD precursor (Iqbal et al., 2010). Long chain fatty acid binding and phosphoserine residue binding showed strong results (adjusted p-value = 0.04964 for both, combined scores = 828.65), suggesting metabolic and post-translational monitoring dysregulation. In the case of mammalian phenotype level, the abnormal peripheral nervous system synaptic transmission with values (adjusted p-value = 0.008775, combined score = 3208.63) and abnormal endplate potential with values (adjusted p-value = 0.01166, combined score = 1658.66) were significant, which is consistent with the impact of AD on the synaptic transmission. Decreased paired-pulse facilitation with values (adjusted p-value = 0.01781, combined score = 961.11) proves impaired synaptic plasticity, further assisting neurodegeneration. From the tissue specific analysis, the cortex of the brain (adjusted p-value = 0.0001872, combined score = 511.81) and the ganglia with values (adjusted p-value = 0.0007525, combined score = 280.10) showed significant involvement, which are shown to be critically affected in AD. Within the disease association, strong links were noticed related to brain disease, pineoblastoma, dumping syndrome and dysembryoplastic neuroepithelial tumour, hinting at overlapping pathways of neurodegeneration. The analysis has given a hint related to the metabolic causes of the development of AD, where neuraminidase analogues like sialyltransferase plays a crucial role in disrupting the metabolism of various proteins and lipids. Hence, the enricher-based analysis of highly significant genes has revealed various potential targets for the novel development of therapeutic agents (Table 5).

**TABLE 5:** Results of pre and post MM-GBSA binding free energy components in kcal/mol of the MD simulated molecular complex of Oseltamivir and Neuraminidase.

Energy components	Pre MM-GBSA (Mean ± SD); random seed	Post MM-GBSA (Mean ± SD); random seed	Pre MM-GBSA (Mean ± SD); Seed_1234	Post MM-GBSA (Mean ± SD); Seed_1234
ΔE_vdW (van der Waals)	0.00 ± 0.00	−18.78 ± 0.01	−0.00 ± 0.00	−19.18 ± 0.78
ΔE_elec (Electrostatic)	0.00 ± 0.00	−266.20 ± 0.46	−0.00 ± 0.00	−259.86 ± 1.81
ΔG_polar (Polar solvation, EGB)	242.01 ± 5.04	252.84 ± 0.07	249.12 ± 6.73	250.85 ± 0.52
ΔG_nonpolar (Non-polar solvation, ESURF)	−4.21 ± 0.51	−3.94 ± 0.04	−4.67 ± 0.12	−4.51 ± 0.08
ΔG_gas (Gas-phase energy)	−270.30 ± 3.88	−284.98 ± 0.48	−279.86 ± 3.25	−279.04 ± 1.97
ΔG_solvation (Total solvation)	237.80 ± 5.06	248.91 ± 0.08	244.45 ± 6.73	246.34 ± 0.52
ΔG_bind (Total binding energy)	−32.50 ± 6.38	−36.07 ± 0.49	−35.41 ± 7.47	−32.70 ± 2.04

ΔE_vdW: van der Waals interaction energy between the ligand and the protein.

ΔE_elec: electrostatic interaction energy.

ΔG_polar: solvation free energy due to polar interactions, calculated using the Poisson–Boltzmann (PB) or Generalized Born (GB) models.

ΔG_nonpolar: non-polar solvation free energy, often estimated from the solvent-accessible surface area (SASA).

ΔG_gas: Total energy in vacuum (ΔE_vdW + ΔEEL).

ΔG_solvation: Total solvation energy (ΔG_polar + ΔG_nonpolar).

ΔG_bind: total binding free energy, sum of the above components.

## Discussion

Oseltamivir, which inhibits neuraminidase and facilitates the viral entry into the cells, disarranges sialic acid from the glycoproteins. The sialic acid is also produced by the action of various human enzymes, such as secretases, glycogen synthesis kinases, cycling dependent kinases, protein phosphatase 2A, sialyltransferase, acetylcholinesterase, interleukin 1B and tumour necrosis factor, which plays a vital role either in the formation of insoluble sialic acid or in the production of neurodegeneration through inflammation. Hence, the current study was taken up to analyse the inhibitory potential of oseltamivir against all of the enzymes or cytokines related to Alzheimer’s disorders.

The PASS online tool was used to predict the pharmacological activity of a compound. It was predicted that oseltamivir functions as neuraminidase inhibitor and that it was a preferred antiviral agent against influenza A and B viruses. The ‘Pa’ values exceeding 0.9 were well-aligned with documented role of oseltamivir. A study published by Patricia et al. highlighted the antiviral activity of oseltamivir against influenza (Schirmer and Holodniy, 2009). The findings of this study confirmed the mechanism of action, which helped us in choosing the required topic.

However, the secondary activities such as macrophages stimulation (Hama, 2016) and alpha-N-acetylglucosaminidase inhibition had to settle with lower Pa values, which could be considered as the off-target effects and related to oseltamivir. The secondary activities are not yet well-developed or published, which opens up a potential change of studying the molecular-related activities concerning other diseases for the patient’s safety (Easwaran et al., 2023b).

The toxicity prediction has expressed that the oseltamivir may develop metabolic acidosis (Pa = 0.511), twitching (Pa = 0.517) and dyspnoea (Pa = 0.375). These effects were also noted in the clinical setting with higher doses or with prolonged use of oseltamivir. A published meta-analysis highlights gastrointestinal disturbances and no neuropsychiatric effects of oseltamivir in patients. Such studies confirm the application of oseltamivir with proper dose management (Malosh et al., 2017). Certain predicted toxicities, like thrombophlebitis and ototoxicity, lack substantial clinical evidences. To overcome these limitations of the insilico studies, a necessary clinical validation is recommended. Further, a published study explains that the adverse events related to oseltamivir are milder in intensity, which can be managed clinically (Smith et al., 2011). Nevertheless, the concerns raised by the predictive models as a part of insilico studies cannot be counted less. Hence, a thorough discussion and drug repurposing must be considered (Jhee et al., 2008). In order to avoid the adverse drug reactions or drug interactions of the oseltamivir with those that are milder in action, the drug can be formulated into a lipid or polymer-based carriers as targeted drug. Delivery system implicates its action directly in the targets like neuraminidase analogues or acetylcholinesterase (Farid and Jaffar, 2019; Farid and Jaffar, 2020; Fouad et al., 2017; Jaffar and Farid, 2019) for the application of novel molecules clinically in order to improve the quality of life of those with neurological disorders (Srujana et al., 2017).

The molecular docking studies that employed the PyRx revealed a wide range of binding affinities of oseltamivir to various proteins, included those which are of importance in Alzheimer’s disease. One such target of oseltamivir was acetylcholinesterase (−7.9 kcal/mol), followed by TNF-alpha (−7.4 kcal/mol), GSK-3B (−6.6 kcal/mol) and neuraminidase (−6.5 kcal/mol). These findings suggest the potential off-target interactions of oseltamivir outside its main mechanism of action and antiviral target (Walczak-Nowicka and Herbet, 2021).

Binding affinity to acetylcholinesterase and neuraminidase is particularly important as it highlights the possible role of oseltamivir in controlling cholinergic pathway and neurodegeneration, which explains the involvement of acetylcholinesterase in the development of Alzheimer’s disease (Walczak-Nowicka and Herbet, 2021). The observations of the current study provide high binding affinity of the oseltamivir with acetylcholinesterase; the said action can be validated through the pre-clinical or clinical investigations. Furthermore, the binding affinity of oseltamivir with neuraminidase of −6.5 kcal/mol was consistent with the role of oseltamivir as neuraminidase inhibitor. The observed amino acid interaction with ARG C:172 and GLU A: 128 confirms stable ligand binding aligning with structural studies of Larisa et al (Gubareva and Mohan, 2022) Comparatively, the binding energies with beta and alpha secretase suggest weaker but potentially significant interaction with amyloid precursors protein-processing enzymes. These findings align with the results provided by Kioke et al., that antiviral agents may provide neuroprotective effect through off-target interactions, i.e., sialidase (Hata et al., 2008).

The stability of neuraminidase-oseltamivir complex was confirmed by molecular dynamic simulations studies, as evident RMSD values for Protein: 0.128424–0.493372 nm, ligand 0.122013–0.838708 nm under random seed simulation and for seed_1234 simulation it ranged from Protein 0.161276–2.858013 nm and Ligand 0.208401–3.217241 nm confirming stability of the complex. The result of radius of gyration (for protein at a range of 3.391135–3.518412 nm and for the ligand at a range of 0.354091–0.37452 nm and 3.390434–4.271447 nm and 0.341893–0.371202 nm for seed_1234 simulation) further supports the structural stability of the complex. These findings can be related to the results of a research study published by Putra et al. (2018), which demonstrated similar RMSD for the neuraminidase inhibition forming protein-ligand complex. These results are similar with other earlier published computational studies, affirming strong interaction of oseltamivir with the primary target, i.e., neuraminidase. Similarly, the RMSF values P: 0.0451–0.2189 nm, L: 0.0153–0.1925 nm for random simulation and P: 1.2986–2.2136 nm and ligand L: 0.0160–0.2210 nm under seed_1234 explains extensive molecular interactions, which further validates the oseltamivir’s potency as neuraminidase inhibitor (Abdullahi et al., 2023).

Moreover, with regards to the acetylcholinesterase, the binding energies (-7.9 kcal/mol) also stands as an add-on to the off-target effects of oseltamivir. Combination of inhibition of neuraminidase and acetylcholinesterase in humans may prove to be beneficial in the management of neurological disorders like Alzheimer’s disease (Khunnawutmanotham et al., 2024).

Analysis of the results obtained post MD simulation MM-PBSA and MM-GBSA revealed increased binding affinities of the oseltamivir with Neuraminidase complex. Important energy components such as van der Waals (ΔE_vdW), electrostatic (ΔE_elec) interactions have shown a shift form the negligible (0 ± 0.00) to a favourable value in both productions and trajectories. Which indicates a stronger ligand-protein interactions post MD simulations. On the other hand, polar solvent energy (ΔG_polar) was identified with variable changes, and non-polar energies remains consistently constant. The total binding energies (ΔG_bind) became significantly favourable in random as well as seed_1234 simulations, which confirms improved complex stability. Decomposition level MM-GBSA analysis highlighted critical moieties like GLU:376, GLU:277 and LIG:469 with great shifts in the residual energies of ARG224 and ARG292 post MD simulations. These finding emphasize the value of MD simulations in capturing realistic energetic and structural refinements in ligand binding (Tables 5, 6).

**TABLE 6:** Results of pre and post MM-GBSA binding free energy components in kcal/mol of the MD simulated molecular complex of Oseltamivir and Neuraminidase.

Residue	Random seed pre-simulation results		Random seed post-simulation results		Seed_1234 pre-simulation results		Seed_1234 post-simulation results	
	Total Avg.	Total Std. Dev.	Total Avg.	Total Std. Dev.	Total Avg.	Total Std. Dev.	Total Avg.	Total Std. Dev.
ARG:118	0.160709673	20.6970533	0.073136364	10.86711143	0.4862628	12.27216531	0.160709673	20.6970533
GLU:119	0.145217055	16.97288771	0.312664145	19.51603618	0.0962056	13.84325406	0.145217055	16.97288771
LEU:134	−0.100363636	5.004673121	−0.050090909	5.228447377	−0.0945	4.94543138	−0.100363636	5.004673121
GLN:136	−0.000954545	7.763218447	0.0045	5.5218969	0.018	3.367739887	−0.000954545	7.763218447
ASP:151	0.298093891	8.950598719	0.215914473	7.73179094	1.0444396	4.99261937	0.298093891	8.950598719
ARG:152	−1.308884473	13.31951908	0.528265964	14.93811591	−1.7658424	4.183743335	−1.308884473	13.31951908
ARG:156	0.177119345	11.31163594	0.100202145	17.29397933	−0.053198	10.6508142	0.177119345	11.31163594
TRP:178	−0.781931927	6.321986742	−0.316781782	4.945035206	−0.779634	1.907511047	−0.781931927	6.321986742
SER:179	−0.177992	5.067683233	−0.252709673	7.109482835	−0.2032008	6.822918681	−0.177992	5.067683233
ALA:180	−0.126545455	3.673033655	−0.058454545	4.181619527	−0.118	5.358094251	−0.126545455	3.673033655
ILE:194	−0.217727273	5.611584371	−0.128136364	4.297757474	−0.151	4.03092049	−0.217727273	5.611584371
ILE:222	−0.806077745	4.719285829	−1.333992982	5.225018381	−0.7242256	4.429854294	−0.806077745	4.719285829
LEU:223	0.037590909	4.216363729	0.062454545	3.445386751	0.066	1.001068429	0.037590909	4.216363729
ARG:224	1.984467455	11.9544286	2.420310036	10.85285635	0.9764916	4.983251277	1.984467455	11.9544286
THR:225	−0.315034182	5.214389646	−0.096469236	5.560143728	−0.039	3.686086622	−0.315034182	5.214389646
GLU:227	−1.129660982	16.40106878	0.251115018	12.70363795	−0.6225964	5.087889271	−1.129660982	16.40106878
SER:246	0.017864982	16.35876677	0.086831455	7.270088086	0.0887052	2.333831796	0.017864982	16.35876677
ASN:247	−0.007402909	6.693001681	−0.015727273	5.982405279	0.0077264	13.05948338	−0.007402909	6.693001681
HIS:274	−0.036954545	6.725702928	−11.95182465	9.375090448	−0.0515	3.514541713	−0.036954545	6.725702928
GLU:276	−10.35734345	10.12830086	−15.6124636	13.24161797	−10.5700584	3.694668202	−10.35734345	10.12830086
GLU:277	−11.97835425	9.960964089	3.874111455	13.02749532	−11.2034756	12.73485215	−11.97835425	9.960964089
ARG:292	3.614850218	12.26243563	−0.4706028	10.75649373	2.5030608	14.28342655	3.614850218	12.26243563
ASN:294	0.026062909	8.481015303	−0.226556036	7.736542115	−1.137642	3.21043479	0.026062909	8.481015303
TYR:347	−0.617738473	6.642141102	−0.172151127	4.10205902	−0.954044	6.307583365	−0.617738473	6.642141102
GLY:348	−0.118318182	6.118548282	0.151545455	8.723824196	−0.0255	3.935544276	−0.118318182	6.118548282
ARG:371	0.162681818	11.27644502	0.265398218	17.65578963	0.6629548	5.726531147	0.162681818	11.27644502
TYR:406	−0.048908909	5.856747652	−0.424290836	6.079761746	0.2029712	5.262203844	−0.048908909	5.856747652
PRO:431	−0.0085	4.373965825	−0.014954545	4.81802992	−0.047	1.539013945	−0.0085	4.373965825
LIG:469	−12.39375356	11.25346838	0.019090909	9.532751049	−14.1827488	4.869457648	−12.39375356	11.25346838

The genes, which play a vital role in the TUBB3 (formation of beta-tubulin), FABP3 (regulates alpha-Synuclein uptake in dopaminergic neurons) and CALM1 (Calcium signal transduction pathway) in Alzheimer’s disease, were upregulated. These findings align with the earlier studies published by Shu et al., which reported similar gene expression patterns in AD. The upregulation of neuronal structure and calcium signalling pathways provides further insights into the molecular basics of AD (Wang et al., 2012). Oseltamivir’s potential to modulate the gene expression, particularly in sialyltransferase-related pathways, requires further investigation (Fang et al., 2014). The current study does not directly demonstrate the ligands’ impact on the genes. The observed docking ability of ligand against sialyltransferase (−6.4 kcal/mol) suggests a possible role in controlling glycosylation process related to neurodegeneration. This aligns with the study published by Jannis et al., which highlights the importance of sialylation in brain function and pathological development (Wißfeld et al., 2024) (Tables 7, 8).

**TABLE 7:** Gene expression analysis of sialyltransferase gene with accession number GSE5281.

Accession number	Groups	ID	Adj. P- value	Log FC	Gene symbol	Gene title
GSE5281 Focusing on the Alzheimer’s disease	Control groups: 61 samples Case group: 44 samples	213476_x_at	6.25E-10	1.999	TUBB3	Tubulin beta 3 class III
		202257_s_at	9.34E-10	2.022	CD2BP2	CD2 cytoplasmic tail binding protein 2
		230656_s_at	1.00E-09	2.163	UTP4	UTP4, small subunit processome component
		207232_s_at	1.00E-09	2.027	DZIP3	DAZ interacting zinc finger protein 3
		205738_s_at	5.07E-09	2.028	FABP3	Fatty acid binding protein 3
		213921_at	8.05E-09	2.759	SST	Somatostatin
		202785_at	8.40E-09	2.172	NDUFA7	NADH: ubiquinone oxidoreductase subunit A7
		223708_at	1.30E-08	2.636	C1QTNF4	C1q and tumour necrosis factor related protein 4
		221772_s_at	1.44E-08	1.926	PPP2R2D	Protein phosphatase 2 regulatory subunit B delta
		1568603_at	1.56E-08	2.250	CADPS	Calcium dependent secretion activator
		201387_s_at	1.63E-08	1.956	UCHL1	Ubiquitin C-terminal hydrolase L1
		202507_s_at	1.72E-08	2.319	SNAP25	Synaptosome associated protein 25
		215020_at	1.94E-08	2.147	NRXN3	Neurexin 3
		1568604_a_at	2.08E-08	2.130	CADPS	Calcium dependent secretion activator
		219549_s_at	2.58E-08	2.027	RTN3	Reticulon 3
		213710_s_at	2.84E-08	2.127	CALM1	Calmodulin 1
		200638_s_at	3.00E-08	1.948	YWHAZ	Tyrosine 3-monooxygenase/tryptophan 5-monooxygenase activation protein zeta
		219408_at	3.15E-08	1.989	PRMT7	Protein arginine methyltransferase 7
		215021_s_at	3.44E-08	2.031	NRXN3	Neurexin 3
		209953_s_at	3.92E-08	1.893	CDC37	Cell division cycle 37

**TABLE 8:** The results of significant gene expression from the data of 20 highly upregulated and significantly expressed genes.

Index	Name	P-value	Adjusted p-value	Odds Ratio	Combined score
Cellular components					
1	Vesicle	0.001147	0.04964	17.03	115.27
2	Glutamatergic Synapse	0.002206	0.04964	32.74	200.25
Molecular function					
1	Kinase Binding	0.0006541	0.03205	12.23	89.70
2	Protein Phosphatase Regulator Activity	0.001357	0.03324	42.21	278.70
3	Adenylate Cyclase Regulator Activity	0.004492	0.04964	293.79	1588.07
4	Long-Chain Fatty Acid Binding	0.007179	0.04964	167.86	828.65
5	Phosphoserine Residue Binding	0.007179	0.04964	167.86	828.65
6	Omega Peptidase Activity	0.008072	0.04964	146.87	707.80
Mammalian phenotype level 4					
1	Abnormal PNS Synaptic Transmission	0.00003428	0.008775	312.09	3208.63
2	Abnormal Endplate Potential	0.00009112	0.01166	178.29	1658.66
3	Decreased Paired-Pulse Facilitation	0.0002087	0.01781	113.41	961.11
Jensen tissue					
1	Brain cortex cell line	9.549e-7	0.0001872	36.92	511.81
2	Ganglion	0.000007678	0.0007525	23.78	280.10
3	Interstitial cell of Cajal	0.00002361	0.001543	29.76	317.08
4	Temporal lobe	0.0004218	0.01516	6.88	53.46
5	Parietal lobe	0.0005833	0.01516	8.14	60.61
Jensen diseases					
1	Brain disease	0.002151	0.03929	33.18	203.78
2	Pineoblastoma	0.005388	0.03929	235.02	1227.64
3	Dumping syndrome	0.007179	0.03929	167.86	828.65
4	Dysembryoplastic neuroepithelial tumour	0.007179	0.03929	167.86	828.65

Functional enrichment analysis revealed significant association with molecular function, such as kinase binding (p = 0.0006541) and protein phosphatase regulatory activity (p = 0.001357). These findings explore the role of oseltamivir in modulating signal pathways indirectly through protein interactions. Additionally, the action on the cellular components, such as vesicle and glutaminergic synapse, underlines the potential impact of oseltamivir on synaptic transmission. Furthermore, the association of oseltamivir’s activity with brain cortex cell lines and disease such as dysembryoplastic neuroepithelial tumour (p = 0.007179) raises a point regarding the higher implications of the oseltamivir use in neurological conditions. These findings also align with the earlier published studies by Pamela et al., which reported off-target effects on the neural pathways. However, a thorough experimental validation is still required to justify these activities obtained through *in silico* studies (Ferreira et al., 2020).

## Conclusion

The current study aims to explore the impact of oseltamivir as an inhibitor on neuraminidase and its analogues in humans, along with the significant genes and pathways involved in the AD. The results highlight the binding affinities of oseltamivir with neuraminidase, acetylcholinesterase and sialyltransferase, along with the importance of synaptic functions, vesicle transport and kinase-regulating genes, suggesting their pivotal role in the progression of AD. The binding affinities, synaptic functions, vesicle transport and kinase regulating are prior expressed in various research publications linking to cognition decline and development of AD. The identified enzymes can be considered as important molecular targets for developing novel drugs to treat AD in humans. However, a small number of target proteins and insilico methods employed in the study needs to be validated experimentally. Future research anchoring on the same idea should focus on the understanding of molecular mechanisms and clinical validations to solidify the findings with the therapeutic potential of these targets in AD. The current study provides a deeper understanding of the various patterns related to AD, which could help in the future development of novel agents.

## Limitations and future directions

This study provides comprehensive yet limited insights regarding oseltamivir’s pharmacology and toxicology profile. The insilico predictions and docking studies require experimental validations to confirm their applicability in clinical settings. Additionally, the observed gene expression analysis and pathway association are correlative and do not establish causality.

Future studies should focus on experimental validation of the pharmacological and toxicological activities predicted related to oseltamivir, along with its off-target interaction. For oseltamivir repurposing, both *in vitro* and *in vivo* studies are required to confirm its effect on the neurological pathways and to correct the neurological diseases. Additionally, integrating multi-omics data such as proteomics and metabolomics could possibly elucidate the molecular mechanisms highlighting oseltamivir’s pharmacological effects. However, through this study, the potential pharmacological, toxicological and gene regulatory activities of the oseltamivir are uncovered as neuraminidase inhibitor. Likewise, the potential secondary activities and off-target interactions are also uncovered. These findings provide a broader therapeutic applications and safety profile of oseltamivir. Moreover, experimental validation is being considered in collaboration with renowned research institutes and we hope to explore this aspect in the upcoming series works.

## Data Availability

The data can be found in the given link below: https://figshare.com/s/e2b28ab2503735cd6302.

## References

[B1] Abdullahi M. Uzairu A. Shallangwa G. A. Mamza P. A. Ibrahim M. T. Chandra A. (2023). Unveiling 1,3-thiazine derivative as a potential neuraminidase inhibitor: molecular docking, molecular dynamics, ADMET and DFT studies. Chem. Afr. 6 (6), 2957–2967. 10.1007/s42250-023-00713-4

[B2] Alroy J. García-Moliner M. L. Lee R. E. (2014). “The pathology of the skeleton in lysosomal storage diseases,” in Pathobiology of human disease. Editors McManus L. M. Mitchell R. N. (San Diego: Academic Press), 874–892.

[B3] Ashburner M. Ball C. A. Blake J. A. Botstein D. Butler H. Cherry J. M. (2000). Gene Ontology: tool for the unification of biology. Nat. Genet. 25 (1), 25–29. 10.1038/75556 10802651 PMC3037419

[B4] Baazaoui N. Iqbal K. (2022). Alzheimer’s disease: challenges and a therapeutic opportunity to treat it with a neurotrophic compound. Biomolecules 12 (10), 1409. 10.3390/biom12101409 36291618 PMC9599095

[B5] Baraskar K. Thakur P. Shrivastava R. Shrivastava V. K. (2021). Female obesity: association with endocrine disruption and reproductive dysfunction. Obes. Med. 28, 100375. 10.1016/j.obmed.2021.100375

[B6] Baroroh S. S. M. B. U. Muscifa Z. S. Destiarani W. Rohmatullah F. G. Yusuf M. (2023). Molecular interaction analysis and visualization of protein-ligand docking using Biovia Discovery Studio Visualizer. Indonesian J. Comput. Biol. (IJCB) 2 (1), 22. 10.24198/ijcb.v2i1.46322

[B7] Barthold D. Joyce G. Ferido P. Drabo E. F. Marcum Z. A. Gray S. L. (2020). Pharmaceutical treatment for alzheimer's disease and related dementias: utilization and disparities. J. Alzheimers Dis. 76 (2), 579–589. 10.3233/jad-200133 32538845 PMC7825250

[B8] Bekker H. Berendsen H. J. C. Dijkstra E. J. Achterop S. Vondrumen R. Vanderspoel D. (1993). “Gromacs - a parallel computer for molecular-dynamics simulations,” in 4th international conference on computational physics (PC 92). Editors DeGroot R. A. Nadrchal J. (World Scientific Publishing: SINGAPORE), 252–256.

[B9] Berman H. M. Westbrook J. Feng Z. Gilliland G. Bhat T. N. Weissig H. (2000). The protein data bank. Nucleic Acids Res. 28 (1), 235–242. 10.1093/nar/28.1.235 10592235 PMC102472

[B10] Bird N. L. Olson M. R. Hurt A. C. Oshansky C. M. Oh D. Y. Reading P. C. (2015). Oseltamivir prophylaxis reduces inflammation and facilitates establishment of cross-strain protective T cell memory to influenza viruses. PLOS ONE 10 (6), e0129768. 10.1371/journal.pone.0129768 26086392 PMC4473273

[B11] Bishop P. Rocca D. Henley J. M. (2016). Ubiquitin C-terminal hydrolase L1 (UCH-L1): structure, distribution and roles in brain function and dysfunction. Biochem. J. 473 (16), 2453–2462. 10.1042/bcj20160082 27515257 PMC4980807

[B12] Breijyeh Z. Karaman R. (2020). Comprehensive review on Alzheimer’s disease: causes and treatment. Molecules 25 (24), 5789. 10.3390/molecules25245789 33302541 PMC7764106

[B13] Bugnon M. Goullieux M. Röhrig U. F. Perez M. A. S. Daina A. Michielin O. (2023). SwissParam 2023: a modern web-based tool for efficient small molecule parametrization. J. Chem. Inf. Model. 63 (21), 6469–6475. 10.1021/acs.jcim.3c01053 37853543 PMC10649791

[B14] Bytheway I. Darley M. G. Popelier P. L. A. (2008). The calculation of polar surface area from first principles: an application of quantum chemical topology to drug design. ChemMedChem 3 (3), 445–453. 10.1002/cmdc.200700262 18161739

[B15] Carroll C. M. Li Y.-M. (2016). Physiological and pathological roles of the γ-secretase complex. Brain Res. Bull. 126, 199–206. 10.1016/j.brainresbull.2016.04.019 27133790 PMC5436903

[B16] Chahinian H. Yahi N. Fantini J. (2024). Glutamate, gangliosides, and the synapse: electrostatics at work in the brain. Int. J. Mol. Sci. 25 (16), 8583. 10.3390/ijms25168583 39201269 PMC11354842

[B17] Chen E. Y. Tan C. M. Kou Y. Duan Q. Wang Z. Meirelles G. V. (2013). Enrichr: interactive and collaborative HTML5 gene list enrichment analysis tool. BMC Bioinforma. 14 (1), 128. 10.1186/1471-2105-14-128 PMC3637064 23586463

[B18] Chen S. Cao Z. Nandi A. Counts N. Jiao L. Prettner K. (2024). The global macroeconomic burden of Alzheimer's disease and other dementias: estimates and projections for 152 countries or territories. Lancet Glob. Health 12 (9), e1534–e1543. 10.1016/s2214-109x(24)00264-x 39151988

[B19] Chin D. Means A. R. (2000). Calmodulin: a prototypical calcium sensor. Trends Cell Biol. 10 (8), 322–328. 10.1016/s0962-8924(00)01800-6 10884684

[B20] Corbo C. P. Alonso A. d.C. (2011). “Chapter 2 - therapeutic targets in alzheimer's disease and related tauopathies,” in Progress in molecular biology and translational science. Editor Rahman S. (Academic Press), 47–83. 10.1016/B978-0-12-385506-0.00002-8 21199770

[B21] Coutsias E. A. Wester M. J. (2019). RMSD and symmetry. J. Comput. Chem. 40 (15), 1496–1508. 10.1002/jcc.25802 30828834

[B22] Dallakyan S. Olson A. J. (2015). “Small-molecule library screening by docking with PyRx,” in Chemical biology: methods and protocols. Editors Hempel J. E. Williams C. H. Hong C. C. (New York, NY: Springer New York), 243–250. 10.1007/978-1-4939-2269-7_19 25618350

[B23] DeTure M. A. Dickson D. W. (2019). The neuropathological diagnosis of Alzheimer’s disease. Mol. Neurodegener. 14 (1), 32. 10.1186/s13024-019-0333-5 31375134 PMC6679484

[B24] Du J. Shui H. Chen R. Dong Y. Xiao C. Hu Y. (2024). Neuraminidase-1 (NEU1): biological roles and therapeutic relevance in human disease. Curr. Issues Mol. Biol. 46 (8), 8031–8052. 10.3390/cimb46080475 39194692 PMC11353077

[B25] Duly A. M. P. Kao F. C. L. Teo W. S. Kavallaris M. (2022). Βiii-tubulin gene regulation in health and disease. Front. Cell Dev. Biol. 10, 851542. 10.3389/fcell.2022.851542 35573698 PMC9096907

[B26] Dzulko M. Pons M. Henke A. Schneider G. Krämer O. H. (2020). The PP2A subunit PR130 is a key regulator of cell development and oncogenic transformation. Biochimica Biophysica Acta (BBA) - Rev. Cancer 1874 (2), 188453. 10.1016/j.bbcan.2020.188453 33068647

[B27] Easwaran V. Almeleebia T. M. Mantargi M. J. S. Khan N. A. Alshahrani S. M. Orayj K. (2023b). Patient safety culture in the southern region of Saudi arabia: a survey among community pharmacies. Healthcare 11 (10), 1416. 10.3390/healthcare11101416 37239699 PMC10218386

[B28] Easwaran V. Khan N. A. Iqbal M. J. Alshahrani S. M. Orayj K. Almeleebia T. M. (2023a). The study of healthcare professionals' perspective towards the quality of diabetic care services in Abha. Eur. Rev. Med. Pharmacol. Sci. 27 (10), 4328–4336. 10.26355/eurrev_202305_32437 37259764

[B29] Fang Q. Gao Y. Chen M.-f. Guo X.-l. Yang X. Yang X. (2014). Molecular epidemiology and evolution of A(H1N1)pdm09 and H3N2 virus during winter 2012–2013 in Beijing, China. J. Mol. Epidemiol. Evol. Genet. Infect. Dis. 26, 228–240. 10.1016/j.meegid.2014.05.034 24911284

[B30] Farid M. Jaffar M. (2019). Creation and assessment of buccal mucoadhesive sustained release oral films. Int. J. Pharm. Pharm. Res. 16 (2), 30–41.

[B31] Farid M. Jaffar M. (2020). Formulation and evaluation of ofloxacin loaded chitosan nanoparticles. WORLD J. Pharm. Pharm. Sci. 9 (10), 1–13.

[B32] Ferreira P. C. L. De Bastiani M. A. Bellaver B. Povala G. Brum W. S. Ramos V. G. (2020). Functional enrichment analysis of differentially expressed genes leads to dysregulation in biological processes networks in Alzheimer’s disease continuum. Alzheimer's & Dementia 16 (S2), e039506. 10.1002/alz.039506

[B33] Forsyth E. Ritzline P. D. (1998). An overview of the etiology, diagnosis, and treatment of alzheimer disease. Phys. Ther. 78 (12), 1325–1331. 10.1093/ptj/78.12.1325 9859951

[B34] Fouad M. Zaki A. Shabana S. El-Haggar R. Haggar M. Jaffar S. (2017). Evaluation of oral hypoglycemic potency of Medicago polymorpha and Zygophyllum simplex: a Drug-Drug interaction study. Medicine 6 (6), 648–651.

[B35] Gao C. Jiang J. Tan Y. Chen S. (2023). Microglia in neurodegenerative diseases: mechanism and potential therapeutic targets. Signal Transduct. Target. Ther. 8 (1), 359. 10.1038/s41392-023-01588-0 37735487 PMC10514343

[B36] Germoush M. O. Mantargi M. J. S. Sarhan M. Alrashdi B. M. Massoud D. Alzwain S. (2024). Molecular docking of eleven snake venom peptides targeting human immunodeficiency virus capsid glycoprotein as inhibitors. Open Veterinary J. 14 (11), 2936–2949. 10.5455/ovj.2024.v14.i11.22 PMC11682769 39737016

[B37] Glanz V. Y. Myasoedova V. A. Grechko A. V. Orekhov A. N. (2018). Inhibition of sialidase activity as a therapeutic approach. Drug Des. Devel Ther. 12, 3431–3437. 10.2147/dddt.s176220 PMC6186905 30349196

[B38] Green M. D. Nettey H. Wirtz R. A. (2008). Determination of oseltamivir quality by colorimetric and liquid chromatographic methods. Emerg. Infect. Dis. J. 14 (4), 552–556. 10.3201/eid1404.061199 PMC2570928 18394271

[B39] Grumati P. Morozzi G. Hölper S. Mari M. Harwardt M.-L. I. E. Yan R. (2017). Full length RTN3 regulates turnover of tubular endoplasmic reticulum via selective autophagy. eLife 6, e25555. 10.7554/elife.25555 28617241 PMC5517149

[B40] Gubareva L. Mohan T. (2022). Antivirals targeting the neuraminidase. Cold Spring Harb. Perspect. Med. 12 (1), a038455. 10.1101/cshperspect.a038455 32152244 PMC8725622

[B41] Hama R. (2016). The mechanisms of delayed onset type adverse reactions to oseltamivir. Infect. Dis. 48 (9), 651–660. 10.1080/23744235.2016.1189592 PMC4973146 27251370

[B42] Hata K. Koseki K. Yamaguchi K. Moriya S. Suzuki Y. Yingsakmongkon S. (2008). Limited inhibitory effects of oseltamivir and zanamivir on human sialidases. Antimicrob. Agents Chemother. 52 (10), 3484–3491. 10.1128/aac.00344-08 18694948 PMC2565904

[B43] Holtzman D. M. Morris J. C. Goate A. M. (2011). Alzheimer’s disease: the challenge of the second century. Sci. Transl. Med. 3 (77), 77sr1. 10.1126/scitranslmed.3002369 21471435 PMC3130546

[B44] Iqbal K. Liu F Fau - Gong C. X. Gong Cx Fau - Grundke-Iqbal I. Grundke-Iqbal I. (2010). Tau in Alzheimer disease and related tauopathies. Curr. Alzheimer Res. 7 (8), 656–664. 10.2174/156720510793611592 20678074 PMC3090074

[B45] Jaffar M. Farid M. (2019). Formulation and *in vivo* evaluation of risperidone spherical agglomerates prepared for earlier absorption of risperidone. World J. Pharm. Res. 8 (13), 1430–1444.

[B46] Jhee S. S. Yen M. Ereshefsky L. Leibowitz M. Schulte M. Kaeser B. (2008). Low penetration of oseltamivir and its carboxylate into cerebrospinal fluid in healthy Japanese and caucasian volunteers. Antimicrob. Agents Chemother. 52 (10), 3687–3693. 10.1128/aac.00327-08 18676886 PMC2565879

[B47] Kalyan G. U. Reddy D. P. Chandrakanth G. Pooja B. Anitha V. Vivek D. (2023). “Gene association disease prediction by GEO2R tool,” in 2023 international conference on evolutionary algorithms and soft computing techniques (EASCT).

[B48] Keil J. M. Rafn G. R. Turan I. M. Aljohani M. A. Sahebjam-Atabaki R. Sun X.-L. (2022). Sialidase inhibitors with different mechanisms. J. Med. Chem. 65 (20), 13574–13593. 10.1021/acs.jmedchem.2c01258 36252951 PMC9620260

[B49] Kelley B. J. Petersen R. C. (2007). Alzheimer's disease and mild cognitive impairment. Neurol. Clin. 25 (3), 577–609. 10.1016/j.ncl.2007.03.008 17659182 PMC2682228

[B50] Khatua B. Roy S. Mandal C. (2013). Sialic acids siglec interaction: a unique strategy to circumvent innate immune response by pathogens. Indian J. Med. Res. 138 (5), 648–662. 24434319 PMC3928697

[B51] Khunnawutmanotham N. Sooknual P. Batsomboon P. Ploypradith P. Chimnoi N. Patigo A. (2024). Synthesis, antiacetylcholinesterase activity, and molecular dynamics simulation of aporphine–benzylpyridinium conjugates. ACS Med. Chem. Lett. 15 (1), 132–142. 10.1021/acsmedchemlett.3c00467 38229749 PMC10788943

[B52] Kim S. Chen J. Cheng T. Gindulyte A. He J. He S. (2024). PubChem 2025 update. Nucleic Acids Res. 53, D1516–D1525. 10.1093/nar/gkae1059 PMC11701573 39558165

[B53] Kuleshov M. V. Jones M. R. Rouillard A. D. Fernandez N. F. Duan Q. Wang Z. (2016). Enrichr: a comprehensive gene set enrichment analysis web server 2016 update. Nucleic Acids Res. 44 (W1), W90–W97. 10.1093/nar/gkw377 27141961 PMC4987924

[B54] Lee S.-M. Lee S. H. Jung Y. Lee Y. Yoon J. H. Choi J. Y. (2020). FABP3-mediated membrane lipid saturation alters fluidity and induces ER stress in skeletal muscle with aging. Nat. Commun. 11 (1), 5661. 10.1038/s41467-020-19501-6 33168829 PMC7653047

[B55] Liang W. S. Dunckley T. Beach T. G. Grover A. Mastroeni D. Ramsey K. (2008b). Altered neuronal gene expression in brain regions differentially affected by Alzheimer's disease: a reference data set. Physiol. Genomics 33 (2), 240–256. 10.1152/physiolgenomics.00242.2007 18270320 PMC2826117

[B56] Liang W. S. Dunckley T. Beach T. G. Grover A. Mastroeni D. Walker D. G. (2007). Gene expression profiles in anatomically and functionally distinct regions of the normal aged human brain. Physiol. Genomics 28 (3), 311–322. 10.1152/physiolgenomics.00208.2006 17077275 PMC2259385

[B57] Liang W. S. Reiman E. M. Valla J. Dunckley T. Beach T. G. Grover A. (2008a). Alzheimer's disease is associated with reduced expression of energy metabolism genes in posterior cingulate neurons. Proc. Natl. Acad. Sci. 105 (11), 4441–4446. 10.1073/pnas.0709259105 18332434 PMC2393743

[B58] Livingston G. Huntley J. Sommerlad A. Ames D. Ballard C. Banerjee S. (2020). Lancet . Lancet 396 (10248), 413–446. 10.1016/s0140-6736(20)30367-6 32738937 PMC7392084

[B59] Magesh S. Savita V. Moriya S. Suzuki T. Miyagi T. Ishida H. (2009). Human sialidase inhibitors: design, synthesis, and biological evaluation of 4-acetamido-5-acylamido-2-fluoro benzoic acids. Bioorg. & Med. Chem. 17 (13), 4595–4603. 10.1016/j.bmc.2009.04.065 19450982

[B60] Magesh S. Suzuki T. Miyagi T. Ishida H. Kiso M. (2006). Homology modeling of human sialidase enzymes NEU1, NEU3 and NEU4 based on the crystal structure of NEU2: hints for the design of selective NEU3 inhibitors. J. Mol. Graph. Model. 25 (2), 196–207. 10.1016/j.jmgm.2005.12.006 16427342

[B61] Malosh R. E. Martin E. T. Heikkinen T. Brooks W. A. Whitley R. J. Monto A. S. (2017). Efficacy and safety of oseltamivir in children: systematic review and individual patient data meta-analysis of randomized controlled trials. Clin. Infect. Dis. 66 (10), 1492–1500. 10.1093/cid/cix1040 29186364

[B62] Maurice P. Baud S. Bocharova O. V. Bocharov E. V. Kuznetsov A. S. Kawecki C. (2016). New insights into molecular organization of human neuraminidase-1: transmembrane topology and dimerization ability. Sci. Rep. 6 (1), 38363. 10.1038/srep38363 27917893 PMC5137157

[B63] McAuley J. L. Gilbertson B. P. Trifkovic S. Brown L. E. McKimm-Breschkin J. L. (2019). Influenza virus neuraminidase structure and functions. Front. Microbiol. 10, 39. 10.3389/fmicb.2019.00039 30761095 PMC6362415

[B64] McKimm-Breschkin J. L. (2013). Influenza neuraminidase inhibitors: antiviral action and mechanisms of resistance. Influenza Other Respir. Viruses 7 (s1), 25–36. 10.1111/irv.12047 23279894 PMC4942987

[B65] Miller B. R. III McGee T. D. Jr. Swails J. M. Homeyer N. Gohlke H. Roitberg A. E. (2012). MMPBSA.py: an efficient program for end-state free energy calculations. J. Chem. Theory Comput. 8 (9), 3314–3321. 10.1021/ct300418h 26605738

[B66] Miyagi T. (2008). “Aberrant expression of sialidase in cancer and diabetes,” in Experimental glycoscience: glycobiology. Editor Taniguchi N., (Tokyo: Springer Japan), 329–332.

[B67] Mu M. Sun H. Geng S. Xu T. Sun C. Zhang Z. (2024). Neurexin-3 in the paraventricular nucleus of the hypothalamus regulates body weight and glucose homeostasis independently of food intake. Mol. Brain 17 (1), 49. 10.1186/s13041-024-01124-3 39090731 PMC11295692

[B68] Murugan N. A. Podobas A. Gadioli D. Vitali E. Palermo G. Markidis S. (2022). A review on parallel virtual screening softwares for high-performance computers. Pharmaceuticals 15 (1), 63. 10.3390/ph15010063 35056120 PMC8780228

[B69] Nichols E. Steinmetz J. D. Vollset S. E. Fukutaki K. Chalek J. Abd-Allah F. (2022). Estimation of the global prevalence of dementia in 2019 and forecasted prevalence in 2050: an analysis for the Global Burden of Disease Study 2019. Lancet Public Health 7 (2), e105–e125. 10.1016/s2468-2667(21)00249-8 34998485 PMC8810394

[B70] O'Boyle N. M. Banck M. James C. A. Morley C. Vandermeersch T. Hutchison G. R. (2011). Open Babel: an open chemical toolbox. J. Cheminformatics 3 (1), 33. 10.1186/1758-2946-3-33 PMC3198950 21982300

[B71] Olsson B. Zetterberg H. Hampel H. Blennow K. (2011). Biomarker-based dissection of neurodegenerative diseases. Prog. Neurobiol. 95 (4), 520–534. 10.1016/j.pneurobio.2011.04.006 21524681

[B72] Partonen T. (2023). “Medication effects,” in Encyclopedia of sleep and circadian rhythms. Editor Kushida C. A. Second Edition) (Oxford: Academic Press), 755–760.

[B73] Passeri E. Elkhoury K. Morsink M. Broersen K. Linder M. Tamayol A. (2022). Alzheimer’s disease: treatment strategies and their limitations. Int. J. Mol. Sci. 23 (22), 13954. 10.3390/ijms232213954 36430432 PMC9697769

[B74] Poroikov V. Filimonov D. Lagunin A. Gloriozova T. Zakharov A. (2007). PASS: identification of probable targets and mechanisms of toxicity†. SAR QSAR Environ. Res. 18 (1-2), 101–110. 10.1080/10629360601054032 17365962

[B75] Puigdellívol M. Allendorf D. H. Brown G. C. (2020). Sialylation and galectin-3 in microglia-mediated neuroinflammation and neurodegeneration. Front. Cell. Neurosci. 14, 162. 10.3389/fncel.2020.00162 32581723 PMC7296093

[B76] Putra R. Imaniastuti R. Nasution M. Kerami D. Tambunan U. (2018). “Molecular docking simulation of neuraminidase influenza A subtype H1N1 with potential inhibitor of disulfide cyclic peptide (DNY, NNY, LRL),” in IOP conference series: materials science and engineering (Bristol, United Kingdom: IOP Publishing).

[B77] Rathod R. Kale A. Joshi S. (2016). Novel insights into the effect of vitamin B12 and omega-3 fatty acids on brain function. J. Biomed. Sci. 23 (1), 17. 10.1186/s12929-016-0241-8 26809263 PMC4727338

[B78] Readhead B. Haure-Mirande J.-V. Funk C. C. Richards M. A. Shannon P. Haroutunian V. (2018). Multiscale analysis of independent Alzheimer’s cohorts finds disruption of molecular, genetic, and clinical networks by human herpesvirus. Neuron 99 (1), 64–82.e7. 10.1016/j.neuron.2018.05.023 29937276 PMC6551233

[B79] Reddy Y. P. Chandrasekhar K. B. Mohammed Jaffar S. (2015). A study of nigella sativa induced growth inhibition of MCF and HepG2 cell lines: an anti-neoplastic study along with its mechanism of action. Pharmacogn. Res. 7 (2), 193. 10.4103/0974-8490.150541 PMC4357971 25829794

[B80] Sambamurti K. (2007). “Beta secretase,” in xPharm: the comprehensive pharmacology reference. Editors Enna S. J. Bylund D. B. (New York: Elsevier), 1–8.

[B81] Samson W. K. Zhang J. V. Avsian-Kretchmer O. Cui K. Yosten G. L. C. Klein C. (2008). Neuronostatin encoded by the somatostatin gene regulates neuronal, cardiovascular, and metabolic functions. J. Biol. Chem. 283 (46), 31949–31959. 10.1074/jbc.m804784200 18753129 PMC2581552

[B82] Sasumana J. Kaushik V. (2018). “Molecular dynamics simulation by using NAMD-VMD and gromacs,” in Journal of emerging technologies and innovative research.

[B83] Schirmer P. Holodniy M. (2009). Oseltamivir for treatment and prophylaxis of influenza infection. Expert Opin. Drug Saf. 8 (3), 357–371. 10.1517/14740330902840519 19355841

[B84] Schütze S. Machleidt T. Krönke M. (1992). Mechanisms of tumor necrosis factor action. Semin. Oncol. 19 (2 Suppl. 4), 16–24. 1313193

[B85] Seo N. Lee H. Oh M. J. Kim G. H. Lee S. G. Ahn J. K. (2021). Isomer-specific monitoring of sialylated N-glycans reveals association of α2,3-linked sialic acid epitope with behcet’s disease. Front. Mol. Biosci. 8, 778851. 10.3389/fmolb.2021.778851 34888356 PMC8650305

[B86] Singh M. K. Shin Y. Ju S. Han S. Kim S. S. Kang I. (2024). Comprehensive overview of Alzheimer’s disease: etiological insights and degradation strategies. Int. J. Mol. Sci. 25 (13), 6901. 10.3390/ijms25136901 39000011 PMC11241648

[B87] Smith J. R. Rayner C. R. Donner B. Wollenhaupt M. Klumpp K. Dutkowski R. (2011). Oseltamivir in seasonal, pandemic, and avian influenza: a comprehensive review of 10-years clinical experience. Adv. Ther. 28 (11), 927–959. 10.1007/s12325-011-0072-7 22057727 PMC7101998

[B88] Srujana M. P. Vigneshwaran E. Kumar G. S. Jyoshna K. Jaffar Sadiq M. J. (2017). Assessment of quality of life in children with epilepsy in rural settings of South India: a cross sectional study. CHRISMED J. Health Res. 4 (2), 110–116. 10.4103/2348-3334.201980

[B89] Tian Z. Feng B. Wang X.-Q. Tian J. (2022). Focusing on cyclin-dependent kinases 5: a potential target for neurological disorders. Front. Mol. Neurosci. 15, 1030639. 10.3389/fnmol.2022.1030639 36438186 PMC9687395

[B90] Treanor J. J. Hayden F. G. Vrooman P. S. Barbarash R. Bettis R. Riff D. (2000). Efficacy and safety of the oral neuraminidase inhibitor oseltamivir in treating acute InfluenzaA randomized controlled trial. JAMA 283 (8), 1016–1024. 10.1001/jama.283.8.1016 10697061

[B91] Varghese A. F. Chockalingam M. P. Viswaksenan S. Santhappan J. S. Ravindran A. L. Balac B. (2024). Experimental determination of radius of gyration of an object using compound pendulum technique. AIP Conf. Proc. 3059 (1), 030005. 10.1063/5.0193845

[B92] Vester S. K. Beavil R. L. Lynham S. Beavil A. J. Cunninghame Graham D. S. McDonnell J. M. (2021). Nucleolin acts as the receptor for C1QTNF4 and supports C1QTNF4-mediated innate immunity modulation. J. Biol. Chem. 296, 100513. 10.1016/j.jbc.2021.100513 33676896 PMC8042453

[B93] Volkow N. D. Ding Y.-S. Fowler J. S. Gatley S. J. (2001). Imaging brain cholinergic activity with positron emission tomography: its role in the evaluation of cholinergic treatments in Alzheimer’s dementia. Biol. Psychiatry 49 (3), 211–220. 10.1016/s0006-3223(00)01112-4 11230872

[B94] Walczak-Nowicka Ł. J. Herbet M. (2021). Acetylcholinesterase inhibitors in the treatment of neurodegenerative diseases and the role of acetylcholinesterase in their pathogenesis. Int. J. Mol. Sci. 22 (17), 9290. 10.3390/ijms22179290 34502198 PMC8430571

[B95] Wang S. Qaisar U. Yin X. Grammas P. (2012). Gene expression profiling in Alzheimer's disease brain microvessels. J. Alzheimer’s Dis. 31 (1), 193–205. 10.3233/jad-2012-120454 22531426

[B96] Wißfeld J. Abou Assale T. Cuevas-Rios G. Liao H. Neumann H. (2024). Therapeutic potential to target sialylation and SIGLECs in neurodegenerative and psychiatric diseases. Front. Neurology 15, 1330874. 10.3389/fneur.2024.1330874 PMC10961342 38529039

[B97] Xiao M. Yao C. Liu F. Xiang W. Zuo Y. Feng K. (2022). Sialic acid ameliorates cognitive deficits by reducing amyloid deposition, nerve fiber production, and neuronal apoptosis in a mice model of Alzheimer’s disease. NeuroSci 3 (1), 28–40. 10.3390/neurosci3010002 39484667 PMC11523747

[B98] Xie Z. Bailey A. Kuleshov M. V. Clarke D. J. B. Evangelista J. E. Jenkins S. L. (2021). Gene set knowledge discovery with enrichr. Curr. Protoc. 1 (3), e90. 10.1002/cpz1.90 33780170 PMC8152575

[B99] Yesselman J. D. Price D. J. Knight J. L. Brooks Iii C. L. (2012). MATCH: an atom-typing toolset for molecular mechanics force fields. J. Comput. Chem. 33 (2), 189–202. 10.1002/jcc.21963 22042689 PMC3228871

[B100] Yuan S. Chan H. C. S. Hu Z. (2017). Using PyMOL as a platform for computational drug design. WIREs Comput. Mol. Sci. 7 (2), e1298. 10.1002/wcms.1298

[B101] Zhang R. Yip V. L. Y. Withers S. G. (2010). “8.11 - mechanisms of enzymatic glycosyl transfer,” in Comprehensive natural products II. Editors Liu H.-W. Mander L. (Oxford: Elsevier), 385–422.

[B102] Zhang W. Xiao D. Mao Q. Xia H. (2023). Role of neuroinflammation in neurodegeneration development. Signal Transduct. Target. Ther. 8 (1), 267. 10.1038/s41392-023-01486-5 37433768 PMC10336149

[B103] Zhao K.-W. Neufeld E. F. (2000). Purification and characterization of recombinant human α-N-acetylglucosaminidase secreted by Chinese hamster ovary cells. Protein Expr. Purif. 19 (1), 202–211. 10.1006/prep.2000.1230 10833408

[B104] Zhu W. Zhou Y. Guo L. Feng S. (2024). Biological function of sialic acid and sialylation in human health and disease. Cell Death Discov. 10 (1), 415. 10.1038/s41420-024-02180-3 39349440 PMC11442784

[B105] Zoete V. Cuendet M. A. Grosdidier A. Michielin O. (2011). SwissParam: a fast force field generation tool for small organic molecules. J. Comput. Chem. 32 (11), 2359–2368. 10.1002/jcc.21816 21541964

